# New azodyrecins identified by a genome mining-directed reactivity-based screening

**DOI:** 10.3762/bjoc.18.102

**Published:** 2022-08-10

**Authors:** Atina Rizkiya Choirunnisa, Kuga Arima, Yo Abe, Noritaka Kagaya, Kei Kudo, Hikaru Suenaga, Junko Hashimoto, Manabu Fujie, Noriyuki Satoh, Kazuo Shin-ya, Kenichi Matsuda, Toshiyuki Wakimoto

**Affiliations:** 1 Faculty of Pharmaceutical Sciences, Hokkaido University, Sapporo 060-0812, Japan; 2 Technology Research Association for Next Generation Natural Products Chemistry, Tokyo 135-0064, Japan; 3 National Institute of Advanced Industrial Science and Technology (AIST), Tokyo 135-0064, Japan; 4 Japan Biological Informatics Consortium (JBIC), Tokyo 135-0064, Japan; 5 Okinawa Institute of Science and Technology Graduate University, Okinawa, 904-0495, Japan; 6 Global Station for Biosurfaces and Drug Discovery, Global Institution for Collaborative Research and Education, Hokkaido University, Sapporo 060-0812, Japan

**Keywords:** biosynthesis, methyltransferase, natural azoxides, reactivity-based screening, *Streptomyces*

## Abstract

Only a few azoxy natural products have been identified despite their intriguing biological activities. Azodyrecins D–G, four new analogs of aliphatic azoxides, were identified from two *Streptomyces* species by a reactivity-based screening that targets azoxy bonds. A biological activity evaluation demonstrated that the double bond in the alkyl side chain is important for the cytotoxicity of azodyrecins. An in vitro assay elucidated the tailoring step of azodyrecin biosynthesis, which is mediated by the *S*-adenosylmethionine (SAM)-dependent methyltransferase Ady1. This study paves the way for the targeted isolation of aliphatic azoxy natural products through a genome-mining approach and further investigations of their biosynthetic mechanisms.

## Introduction

**Figure 1:**
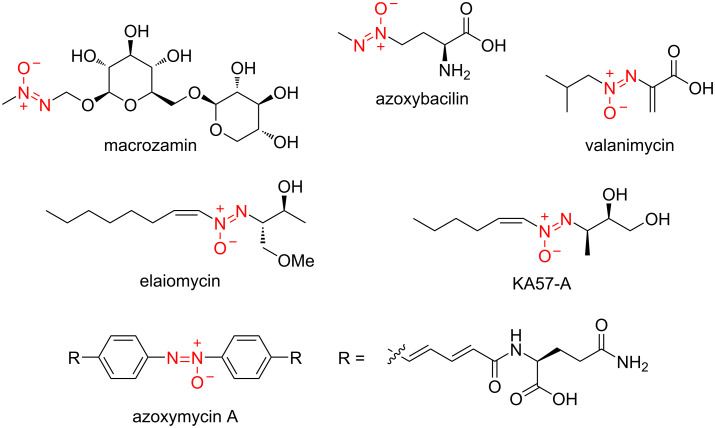
Representative natural azoxides.

Azoxy natural products are a rare yet intriguing class of natural products with various beneficial biological properties, such as antibacterial, antifungal, nematicidal, and cytotoxic activities (Figure 1) [1–3]. Since the discovery of the natural azoxy compound macrozamin in 1951 as the first example of a nitrogen–nitrogen bond-containing natural product [4], azoxy compounds have been isolated from various natural sources including bacteria, fungi, plants, and marine sponges [1–3]. Azoxy natural products have occasionally been discovered by conventional isolation schemes guided by biological activities or physicochemical properties, which are not selective for the azoxy functionality. Consequently, there are only a few examples of azoxy natural products, despite their notable biological activities.

Reactivity-based screening is an emerging strategy in natural products discovery, in which chemical probes are used for the specific detection of the unique functionality of interest in crude metabolites [5–6]. The reactions usually facilitate the subsequent isolation process of the target molecules. This strategy has been successfully applied for detecting a range of peculiar functional groups, such as ureido [7], isocyanide [8], and alkyne [9–10]. A combination of reactivity-based screening and genome-based prioritization would allow the prediction of the producer of natural products with the functional groups of interest, leading to a higher rate of hits. We recently exploited genome mining targeting hydrazine synthetase and the generation of inorganic hydrazine N_2_H_4_ in acid hydrolysate as an indicator of N–N bond-containing functional groups, which led to the discovery of actinopyridazinones with a unique dihydropyridazinone scaffold [11]. N_2_H_4_ generates similarly in the acid hydrolysate of azoxy compounds, and this feature has been exploited as proof for the presence of N–N bonds in the plant-derived methyl azoxy compound macrozamin [4]. Although the N_2_H_4_ generation is an advantageous reactivity of an azoxy group, to the best of our knowledge, it has not been applied for the detection of azoxy bonds in the context of natural products discovery.

The azoxy bond is biosynthesized by two distinct mechanisms. The first is the nitrogen radical coupling mechanism in the biosynthesis of azoxymycins [12–13], which are aromatic azoxy natural products. A similar mechanism has been envisioned for the autoxidation and spontaneous dimerization of aliphatic hydroxylamines via the azoxy linkage in malleobactin D biosynthesis [14]. A distinct mechanism is employed in the biosynthesis of valanimycin, an aliphatic azoxy natural product. This involves the *N*-hydroxylation of isobutylamine, mediated by the flavin-dependent monooxygenase VlmH [15–17], and the following formation of *O*-(ʟ-seryl)-isobutylhydroxylamine by the tRNA-utilizing enzyme VlmA [18]. This intermediate is hypothesized to be transformed into the azoxy bond-containing intermediate via an intramolecular rearrangement accompanied by a concomitant oxidation [18]. Although the exact mechanisms of azoxy bond formation remain unclear, VlmH and VlmA cooperate to biosynthesize the *N*-acyl intermediate for azoxy bonds, suggesting that a genetic region containing both genes could be a potential biosynthetic gene cluster of aliphatic azoxy natural products.

## Results and Discussion

### Reactivity-based isolation of azodyrecins from two *Streptomyces* strains

**Figure 2:**
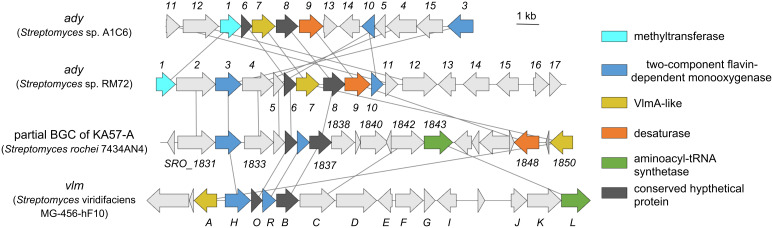
Biosynthetic gene clusters of aliphatic azoxy natural products. Conserved proteins are colored according to their functional annotations. Genes encoding homologous proteins with more than 30% amino acid sequence identities are linked.

**Scheme 1:**
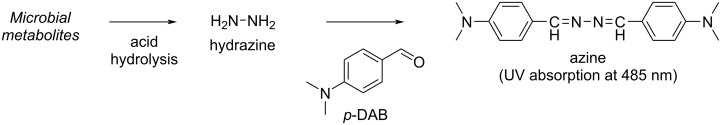
N_2_H_4_-detecting colorimetric assay.

During our efforts toward the discovery of N–N bond-containing natural products from our in-house actinobacterial culture collection, we found two closely related potential biosynthetic gene clusters of aliphatic azoxy natural products in *Streptomyces* sp. RM72 and *Streptomyces* sp. A1C6 (Figure 2 and Tables S1 and S2 in Supporting Information File 1). They encode five genes that are also present in the biosynthetic gene clusters of valanimycin [19] and KA57-A [20]: the putative two-component flavin-dependent monooxygenase similar to VlmH/VlmR, the homologous protein of VlmA, and two additional hypothetical proteins similar to VlmB and VlmO. Based on this observation, we hypothesized that these two *Streptomyces* strains potentially produce aliphatic azoxy natural products similar to valanimycins and KA57-A, and aimed to identify their biosynthetic products. To this end, we conducted a reactivity-based screening to detect N_2_H_4_, which could be generated upon the acid hydrolysis of azoxy natural products. In the assay, N_2_H_4_ is captured by two equivalents of *p*-dimethylaminobenzaldehyde (DAB) to generate *p*-dimethylaminobenzaldazine, which can be sensitively detected by HPLC by monitoring the UV absorption at 485 nm (Scheme 1). As a result, we observed the generation of N_2_H_4_ upon the hydrolysis of solid-culture extracts of both *Streptomyces* strains (Supporting Information File 1, Figure S1). Therefore, we attempted to isolate the azoxy natural products in an N_2_H_4_-detecting assay guided manner.

**Figure 3:**
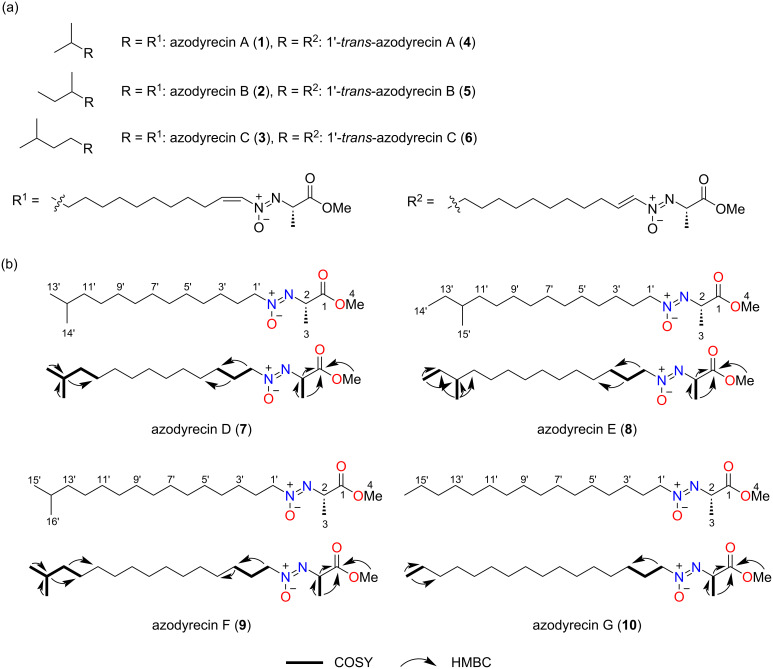
Structures of azodyrecins (a) and new azodyrecin derivatives, azodyrecins D–G (**7**–**10**) (b). Key correlations in 2D NMR are shown.

The extracts of *Streptomyces* sp. RM72 were first partitioned by water and ethyl acetate, and then the organic layer was further fractionated by silica gel column chromatography. Fractionation by reversed-phase HPLC yielded ten compounds (**1**–**10**) that generate N_2_H_4_ upon acid hydrolysis. The combination of ^1^H and ^13^C NMR with a series of 2D NMR analyses and optical rotation revealed that six of the isolated compounds are azodyrecin A (**1**), azodyrecin B (**2**), azodyrecin C (**3**), and their geometric isomers 1’-*trans*-azodyrecin A (**4**), 1’-*trans*-azodyrecin B (**5**), and 1’-*trans*-azodyrecin C (**6**), respectively, which were previously discovered from *Streptomyces* sp. strain P8-A2 (Figure 3a) [21]. Of note, the *trans*-azodyrecins **4**–**6** are reportedly generated by the spontaneous isomerization of the *cis*-congeners, after long-term exposure to CHCl_3_ [21]. While *trans*-azodyrecins could also be generated nonenzymatically from their *cis*-congeners during the isolation process, *trans*-azodyrecins were detected in the fresh extracts of the solid cultures from both strains, suggesting that they are also generated in vivo (Figure S2 in Supporting Information File 1).

The NMR spectra of compound **7** are similar but distinct from those of the known azodyrecins. Close inspection of **7** revealed the absence of olefinic proton signals (7.09 ppm and 6.99 ppm in **4**) and additional methylene proton signals at 4.21 ppm and 1.93 ppm in **7** (Table 1, Figure S3, Supporting Information File 1). The chemical formula predicted with HRMS (C_18_H_36_O_3_N_2_) indicated the presence of two additional hydrogens as compared to compounds **1** and **4**. Collectively, compound **7** was identified as a new analog with a fully saturated alkyl side chain, named azodyrecin D (**7**) (Figure 3b). Likewise, compounds **8**, **9**, and **10** also lack olefinic proton signals (7.09 ppm and 6.99 ppm in **4**) but possess methylene protons (4.21 ppm and 1.93 ppm in **8**, 4.22 ppm and 1.92 ppm in **9**, and 4.21 ppm and 1.81 ppm in **10**). HRMS showed that compound **8** possesses two additional hydrogens compared to **2** and **5**, while compounds **9** and **10** possess two additional hydrogens compared to compounds **3** and **6**. These data, together with 2D NMR spectra showed that compounds **8**, **9**, and **10** were azodyrecin derivatives with saturated alkyl side chains, which were named azodyrecin E (**8**), azodyrecin F (**9**), and azodyrecin G (**10**), respectively (Figure S3, Supporting Information File 1). Although azodyrecins characteristically possess branched alkyl side chains, compound **10** is the only azodyrecin analog with a straight alkyl chain. The configurations of the azoxy groups in **7**–**10** were determined to be *Z*, as in the case of other azodyrecins, according to the characteristic UV absorption at 221 nm (Figure S4 in Supporting Information File 1) [22]. Based on optical rotation values and biosynthetic correlation to known azodyrecins, the configuration of compounds **7**–**10** was defined to be 2*S*.

**Table 1:** ^1^H (500 MHz) and ^13^C (125 MHz) NMR data for azodyrecin D (**7**), azodyrecin E (**8**), azodyrecin F (**9**), and azodyrecin G (**10**).^a^

	compound **7**			compound **8**			compound **9**			compound **10**		

pos.	δ_C_	δ_H_	m, *J* (Hz)	δ_C_	δ_H_	m, *J* (Hz)	δ_C_	δ_H_	m, *J* (Hz)	δ_C_	δ_H_	m, *J* (Hz)

1	172.7			172.7			172.9			170.6		
2	59.9	4.38	q, 7.2	59.9	4.38	q, 7.1	60.0	4.38	q, 7.2	58.1	4.33	q, 7.5
3	16.1	1.45	d, 7.2	16.1	1.45	d, 7.2	15.9	1.45	d, 7.0	15.4	1.38	d, 7.1
4	52.6	3.66	s	52.6	3.67	s	52.5	3.66	s	51.8	3.59	s
1’	70.6	4.21	m	70.6	4.21	m	70.6	4.22	m	68.9	4.21	m
2’	28.9	1.93	m	28.9	1.93	m	28.8	1.92	m	27.2	1.81	m
3’	27.3	1.36^b^	m	27.3	1.37^b^	m	27.2	1.36^b^	m	25.4	1.20–1.30	m
4’	30.2	1.25–1.35	m	30.4	1.25–1.36	m	30.0	1.26–1.36	m	28.3–29.0	1.20–1.30	m
5’	29.2–31.0	1.25–1.35	m	28.4–31.3	1.25–1.36	m	30.5–30.8	1.26–1.36	m	28.3–29.0	1.20–1.30	m
6’	29.2–31.0	1.25–1.35	m	28.4–31.3	1.25–1.36	m	30.5–30.8	1.26–1.36	m	28.3–29.0	1.20–1.30	m
7’	29.2–31.0	1.25–1.35	m	28.4–31.3	1.25–1.36	m	30.5–30.8	1.26–1.36	m	28.3–29.0	1.20–1.30	m
8’	29.2–31.0	1.25–1.35	m	28.4–31.3	1.25–1.36	m	30.5–30.8	1.26–1.36	m	28.3–29.0	1.20–1.30	m
9’	29.2–31.0	1.25–1.35	m	28.4–31.3	1.25–1.36	m	30.5–30.8	1.26–1.36	m	28.3–29.0	1.20–1.30	m
10’	28.7	1.29^b^	m	28.4–31.3	1.25–1.36	m	30.5–30.8	1.26–1.36	m	28.3–29.0	1.20–1.30	m
11’	40.4	1.18	m	37.9	1.31^b^	m	31.0	1.26–1.36	m	28.3–29.0	1.20–1.30	m
			m		1.10^b^	m						m
12’	29.2	1.53	m	35.8	1.33^b^	m	28.5	1.28^b^	m	28.3–29.0	1.20–1.30	m
13’	23.2	0.88	d, 6.7	30.7	1.15	m	40.2	1.17	m	28.3–29.0	1.20–1.30	
					1.25–1.36	m						
14’	23.3	0.88	d, 6.7	12.0	0.87	m	29.1	1.52	m	31.3	1.20–1.30	m
15’				19.9	0.86	m	23.0	0.87	d, 6.7	22.1	1.20–1.30	m
16'							23.0	0.87	d, 6.7	13.9	0.85	d, 6.6

^a^Spectra for compounds **7**–**9** were measured in methanol-*d*_4_ and spectrum for compound **10** was measured in DMSO-*d*_6_; ^b^determined by HSQC.

Fractionation guided by the N_2_H_4_-detecting assay revealed that compounds **1**–**10** were also produced by *Streptomyces* sp. A1C6 (Figure S2 in Supporting Information File 1). This result identified a similar yet distinct type of azodyrecin biosynthetic gene cluster that contains several insertions and inversions, which generally make it challenging to precisely predict its biosynthetic products based solely on genome information (Figure 2). Taken together, the N_2_H_4_-detecting reactivity-based screening led to the identification of four new analogs and two types of biosynthetic gene clusters of azodyrecins, demonstrating its utility in natural product discovery and deorphanization of biosynthetic gene clusters.

### Evaluation of cytotoxicities of azodyrecins

A previous report demonstrated the cytotoxic activity of azodyrecins [21]; however, the relationships between the structure and cytotoxicity were not determined. With new azodyrecin analogs with saturated alkyl chains in hand, we attempted to gain insights into the effects of the double bond on cytotoxicity. To this end, we assessed the cytotoxic activities of azodyrecin B (**2**), 1’-*trans*-azodyrecin B (**5**), together with the most abundant new analogs azodyrecin D (**7**), and azodyrecin E (**8**), against human ovarian adenocarcinoma SKOV-3 cells, malignant pleural mesothelioma MESO-1 cells, immortalized T lymphocyte Jurkat cells, and P388 murine leukemia cells. The results revealed that the derivatives with unsaturated side chains, **2** and **5**, exhibited moderate cytotoxicity against all tested cell lines, with the highest potency against Jurkat cells (IC_50_ at 3.36 μM for **5**) (Table 2 and Figure S5 in Supporting Information File 1). In contrast, the saturated analogs **7** and **8** showed no or negligible cytotoxicity, clearly supporting the importance of the double bond adjacent to the azoxy bond for the cytotoxicity. This result is in contrast to the similar structure–activity relationship (SAR) profiles of elaiomycins, structurally related aliphatic azoxy natural products, in which the antimicrobial and cytotoxic activities do not depend on the double bond adjacent to the azoxy bond [23]. The difference in the SAR profiles between azodyrecins and elaiomycins suggests their distinct modes of actions.

**Table 2:** Cytotoxicities of compound **2**, **5**, **7**, and **8**.

	IC_50_ (µM)			
	
	SKOV-3	MESO-1	Jurkat	P388

azodyrecin B (**2**)	7.37	9.70	8.72	11.6
1’-*trans*-azodyrecin B (**5**)	8.24	6.70	3.36	4.72
azodyrecin D (**7**)	>50	43.2	>50	>50
azodyrecin E (**8**)	>50	>50	>50	>50

### Biosynthetic origin of the unique methyl ester in azodyrecin

**Figure 4:**
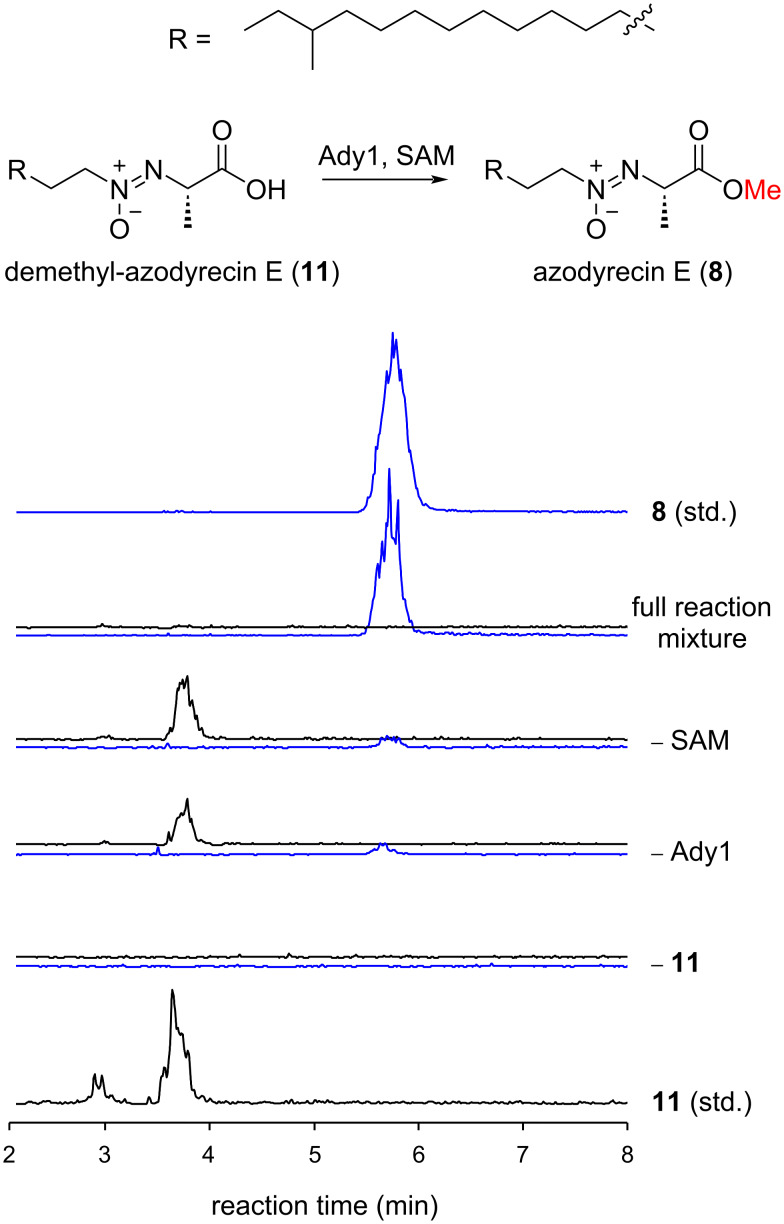
In vitro characterization of Ady1. Extracted ion chromatograms at *m/z* 329.3 (black) and *m/z* 343.3 (blue), corresponding to compound **11** and **8**, respectively, are shown.

The structural diversity of aliphatic azoxy natural products can be attributed to variations in the alkyl side chains and the amino acid-derived counterparts. The variation in the amino acid-derived units is considerably large, as it includes primary and secondary alcohols [24], methoxides [23,25–26], carboxylic acids [27], amides [28], ketones [29–30], an exo-olefin [31], and lactones [32]. Elucidating the mechanisms of structural diversification is essential when considering the synthesis of unnatural azoxides by a synthetic biology-based approach. However, their enzymatic basis has remained elusive except for the exo-olefin formation in valanimycin biosynthesis, which is mediated by the phosphorylation of a serine moiety by VlmJ and the subsequent dehydration by VlmK [33]. To obtain insights into the late-stage diversification mechanisms, we focused on the biosynthesis of the methyl ester, which is unique to azodyrecins. To this end, we characterized the putative SAM-dependent methyltransferase Ady1 in vitro to assess its activity against the carboxylic acid **11**, which was prepared by the hydrolysis of compound **8** under basic conditions. When acid **11** was incubated with recombinant Ady1 in the presence of SAM, it was converted to **8**, showing that Ady1 can install the methyl ester of azodyrecins (Figure 4).

**Scheme 2:**
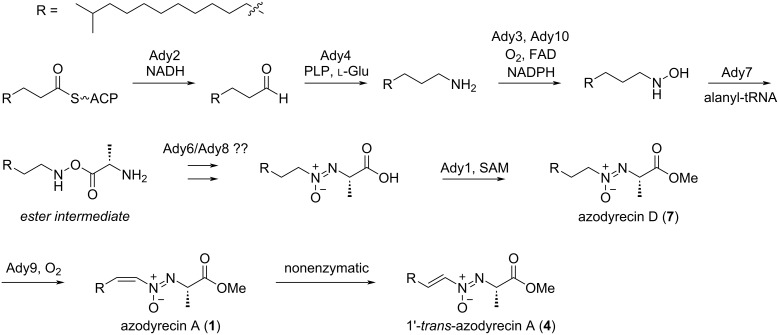
Proposed biosynthetic pathway of azodyrecin.

The in vitro characterization of Ady1 and the functional annotation of *ady* clusters allowed the prediction of the entire biosynthetic pathway of azodyrecins (Scheme 2). The pathway is likely initiated by Ady2, a putative dehydrogenase that recruits fatty acids from primary metabolism to generate an aldehyde, which would be converted to an aliphatic amine by the pyridoxal phosphate (PLP)-dependent transaminase Ady4. The amine would be *N*-hydroxylated by the two-component flavin-dependent monooxygenase Ady3/Ady10, as in the valanimycin biosynthesis mediated by VlmH/VlmR [15–17]. The hydroxylamine would be conjugated to alanyl-tRNA to form an ester intermediate by the function of the tRNA-utilizing enzyme Ady7, which is homologous to VlmA. In valanimycin biosynthesis, the substrate seryl-tRNA is provided by VlmL, an additional seryl-tRNA synthetase (SerRS) encoded within the *vlm* cluster [34]. However, no aminoacyl-tRNA synthetase gene is present in the *ady* cluster, suggesting that the alanyl-tRNA is directly provided from the cellular tRNA pool in the case of azodyrecin biosynthesis. The mechanism for the subsequent rearrangement of the ester intermediate for azoxy bond formation remains unclear; however, the conservation of the two hypothetical proteins Ady6/Ady8 among the biosynthetic gene clusters of valanimycin, KA57-A, and azodyrecins may suggest their participation in this step. Ady6 shows weak homology to DUF4260 (PF14079.9), a family of integral membrane proteins with unknown functions, while Ady8 is similar to the ferritin-like superfamily protein (IPR009078). Ady6/Ady8 are homologous to VlmO/VlmB and SRO_1835/SRO_1837 in the biosynthetic gene clusters of valanimycin and KA57-A, respectively. The in vitro characterization of Ady1 suggested the late-stage biosynthetic pathway of azodyrecin: the azoxy bond formation is followed by the Ady1-mediated methyl esterification to form saturated azodyrecins, and then the subsequent installation of a *cis*-olefin on the 1,2-positions of the alkyl side chain would afford azodyrecins A–C (**1**–**3**), in a reaction likely to be mediated by Ady9, a putative fatty acid desaturase. Nevertheless, the possibility that the desaturation occurs prior to the methyl esterification could not be excluded. The elucidation of the exact order of the late-stage modifications requires further investigations, such as gene knockout experiments and substrate scope analyses of Ady1, which will be accomplished in future work.

### Distribution of potential biosynthetic gene clusters of aliphatic azoxy natural products

**Figure 5:**
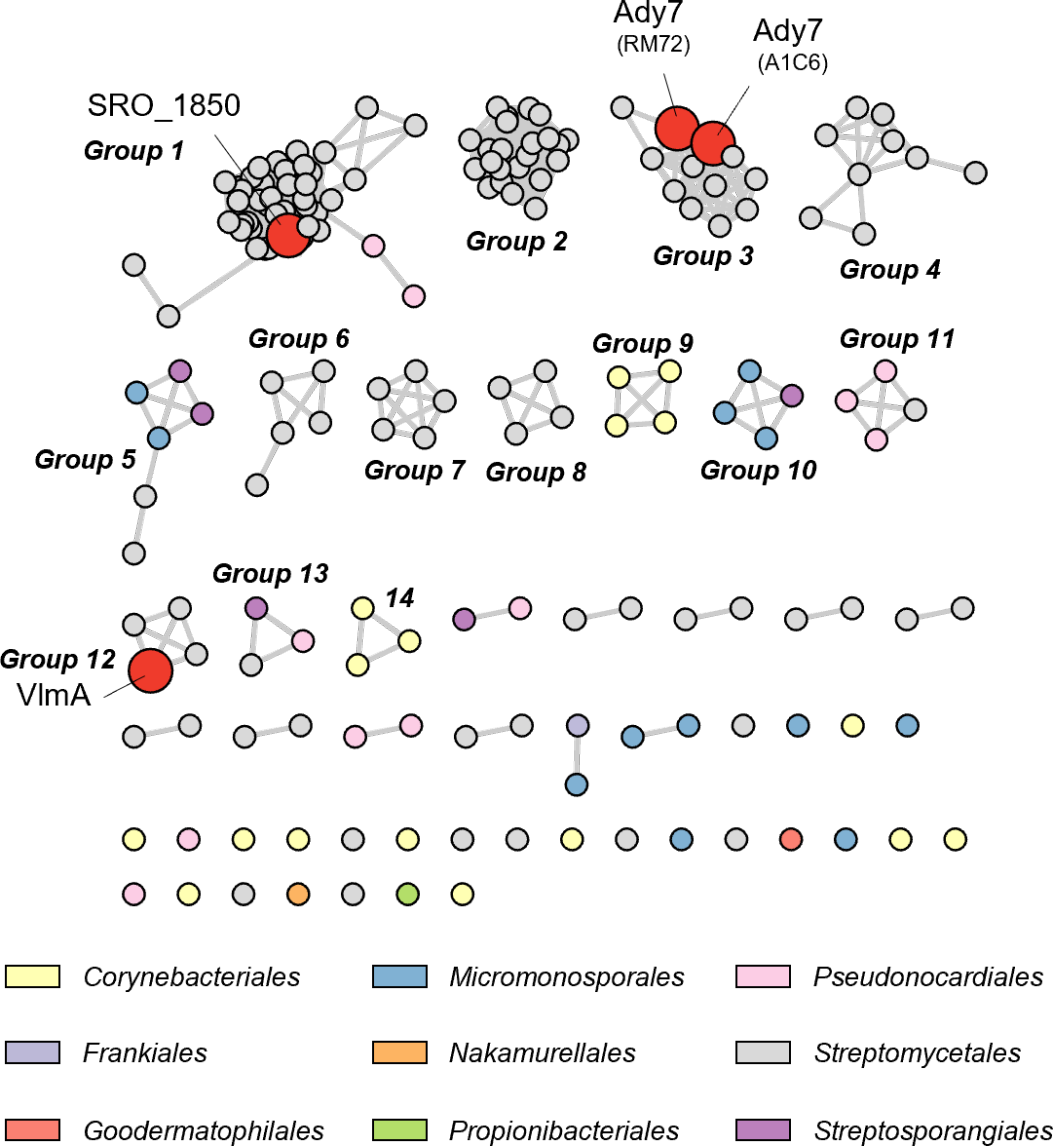
Sequence similarity network of VlmA-like enzymes in the actinobacterial genomes in the Refseq database. Nodes are colored according to the host organism’s order. Enzymes with known biosynthetic products are colored red.

To obtain insights into the distribution and diversity of the biosynthetic gene clusters of aliphatic azoxy natural products, we searched for a pair of VlmA and VlmH, two key enzymes in the azoxy bond formation, encoded in close genetic loci in the reference genome of 3,146 actinobacteria in the NCBI database. A stepwise HMM-based search using models for “VlmA'' (PF09924: LPG_synthase_C) and the amino acid sequence of VlmH identified 179 pairs of VlmA/VlmH, indicating that approximately 5.7% of the actinobacteria present in the NCBI Refseq database are potential producers of aliphatic azoxy natural products. The sequence similarity network (SSN) generated by all-vs-all blastp with *E*-value at 1 × 10^−70^ classified these “VlmAs'' into more than fourteen groups (Figure 5). In this network, the two Ady7s from *Streptomyces* sp. RM72 and *Streptomyces* sp. A1C6 belong to group 3, while VlmA and SRO_1850 belong to groups 12 and 1, respectively. An analysis of the genome neighborhoods of “VlmA” revealed that the three genes encoding “VlmB/O/R” are highly conserved (Figure S7, Supporting Information File 1), suggesting the functional relevance of these genes with VlmA and VlmH. Additionally, a comparison of representative gene clusters from each group indicated that several protein families are frequently observed in the genome neighborhoods of specific “VlmA” groups, such as Ady1-like methyltransferases (PF04072), homologous pairs of VlmJ/VlmK-like exo-olefin-forming enzymes (PF19279/PF03972), seryl-tRNA synthetases (PF02403), putative Trp halogenase-like enzymes (PF04820), and putative 3-oxoacyl-[acyl-carrier-protein (ACP)] synthase III-like enzymes (PF08541) (Figures S7 and S8 in Supporting Information File 1). The various protein families encoded in the proximity of the “VlmA'' gene suggest the manifold biosynthetic pathways and structural diversity of aliphatic azoxy natural products. Considering that most groups in the SSN lack links to their biosynthetic products, a substantial fraction of the chemical diversity in aliphatic natural azoxides likely remains untapped.

## Conclusion

By using the generation of N_2_H_4_ as the indicator of an azoxy bond, we conducted a reactivity-based screening for aliphatic azoxy natural products. This led to the identification of two new producers of azodyrecins, as well as the new analogs **7**–**10**, demonstrating the utility of this reactivity-based strategy for natural products discovery. Bioinformatic surveys shed light on the unexplored biosynthetic potential of actinobacteria for aliphatic azoxides, setting the stage for the targeted isolation of this scarce yet valuable class of natural products with remarkable biological activities.

## Supporting Information

File 1 Experimental procedures, characterization data (^1^H, ^13^C NMR, and HRMS) and biochemical characterization of recombinant Ady1.
