# Supplementary material for: New azodyrecins identified by a genome mining-directed reactivity-based screening
Source: Beilstein J Org Chem. 2022 Aug 10;18:1017–25. doi: 10.3762/bjoc.18.102 (PMC9379638; doi:10.3762/bjoc.18.102)
Supplement: File 1 — Experimental procedures, characterization data (1H, 13C NMR, and HRMS) and biochemical characterization of recombinant Ady1. [file Beilstein_J_Org_Chem-18-1017-s001.pdf]

BEILSTEIN JOURNAL OF ORGANIC CHEMISTRY

Supporting Information

for

New azodyrecins identified by a genome mining-directed reactivity-based screening

Atina Rizkiya Choirunnisa, Kuga Arima, Yo Abe, Noritaka Kagaya, Kei Kudo, Hikaru Suenaga, Junko Hashimoto, Manabu Fujie, Noriyuki Satoh, Kazuo Shin-ya, Kenichi Matsuda and Toshiyuki Wakimoto

Beilstein J. Org. Chem. **2022,** *18,* 1017–1025. doi:10.3762/bjoc.18.102

Experimental procedures, characterization data (^{1}H, ^{13}C NMR, and HRMS) and biochemical characterization of recombinant Ady1

License and Terms: This is a supporting information file under the terms of the Creative Commons Attribution License (https://creativecommons.org/licenses/by/4.0). Please note that the reuse, redistribution and reproduction in particular requires that the author(s) and source are credited and that individual graphics may be subject to special legal provisions.
The license is subject to the *Beilstein Journal of Organic Chemistry* terms and conditions: (https://www.beilstein-journals.org/bjoc/terms)

Table of contents

General remarks

N_2H_4-detecting reactivity-based screening

Isolation of azodyrecins

Cloning and expression of Ady1

In vitro assay of Ady1

Bioinformatics

Supplementary tables

Table S1. Functional annotation of *ady* in *Streptomyces* sp. RM72

Table S2. Functional annotation of *ady* in *Streptomyces* sp. A1C6

Supplementary figures

Figure S1. N_2H_4-detecting colorimetric assay of *Streptomyces* sp. RM72 and *Streptomyces* sp. A1C6.

Figure S2. Production of adozyrecins by *Streptomyces* sp. RM72 and *Streptomyces* sp. A1C6

Figure S3. Comparison of ^{1}H spectra of 1'-*tran*-azodyrecin A (**4**) and new azodyrecin analogs (**7-10**).

Figure S4. UV-Vis spectra of azodyrecins

Figure S5. Cytotoxic assay

Figure S6. SDS-PAGE of Ady1

Figure S7. Protein families that are frequently co-occurring with "VlmA"

Figure S8. Comparison of representative biosynthetic gene clusters of aliphatic azodyrecins

Figure S9–Figure S31. NMR spectra

General remarks. ^{1}H and ^{13}C NMR spectra were recorded on a JEOL ECA500 spectrometer (500 MHz for ^{1}H NMR), a JEOL ECX400P (400 MHz for ^{1}H NMR), a JEOL ECS400 (400 MHz for ^{1}H NMR), a JEOL ECZ400 (400 MHz for ^{1}H NMR) or a Bruker AVANCE Neo (500 MHz for ^{1}H NMR) spectrometer. Chemical shifts are denoted in δ (ppm) relative to residual solvent peaks as internal standard (CD_3OD, ^{1}H δ 3.31, ^{13}C δ 49.0, DMSO-d_6, ^{1}H δ 2.50, ^{13}C δ 39.5). Electrospray ionization mass spectrometry (ESI-MS) spectra were recorded on a Thermo Scientific Exactive mass spectrometer. Liquid chromatography–mass spectroscopy (LC–MS) experiments were performed with a Shimadzu HPLC prominence system coupled with a Shimadzu LCMS-2020 spectrometer or an amaZon SL-NPC system (Bruker Daltonics). All reagents were used as supplied unless otherwise stated. *Escherichia coli* DH5α was used as a host for general cloning. Oligonucleotides used for genetic manipulation were purchased from Fasmac Co.

N_2H_4-detecting reactivity-based screening. 50 μL of crude extracts of actinobacteria were mixed with an equal volume of assay solution containing 10 mM *p*-(dimethylamino)benzaldehyde and 1 M HCl, and incubated at room temperature for 10 min. The resultant mixture was diluted with an equal volume of methanol, centrifuged at 20,630*g* for 10 min, then the supernatant was analyzed by Shimadzu HPLC system equipped with SPD-M20A. 5 μL of the reaction mixture was loaded onto COSMOSIL 5C$_{18}$-MS-II 2.0 × 100 mm (nacalai tesque). The sample was eluted by H_2O/MeCN containing 0.1% formic acid with a linear gradient: 2–98% for MeCN + 0.1% formic acid over 5 min with a flow rate at 0.4 mL/min. Column eluates were monitored by UV absorption at 485 nm.

Isolation of azodyrecins. *Streptomyces* sp. RM72 and *Streptomyces* sp. A1C6 were cultured on YMS^{++} solid media (0.4% yeast extract, 1.0% malt extract, 0.4% soluble starch, after pH was adjusted to 7.4 with KOH solution, 2.0% agar was added. 10 mL of 1 M $MgCl_2$ and 8 mL of $Ca(NO_3)_2$ were added after autoclaving) and SFM solid media (2.0% mannitol, 2.0% soya flour, 2.0% agar), respectively, at 30 °C for 7 days. The resultant media was extracted by methanol, then the residual agar pieces were removed by filtration. Solvents were removed from filtrate, and residues were partitioned between H_2O and ethyl acetate. The organic layer was further partitioned by hexane, then subjected to HP20 column. The column was eluted by a gradient mixture of H_2O/MeOH, then the fractions that generate N_2H_4 upon acid hydrolysis were combined and subjected to silica gel chromatography (40–50 μm silica gel 60N (Kanto Chemical Co.). The column was eluted by a gradient mixture of hexane/$CHCl_3$ then the fractions that generate N_2H_4 upon hydrolysis were combined and the solvent was removed. The crude sample was separated by HPLC with COSMOSIL 5C$_{18}$-MS-II (10 × 250 mm) (nacalai tesque). The column was eluted by H_2O and MeOH by the following conditions: 70–98% in 30 min for MeOH, with a flow rate at 3.0 mL/min. Column elutes were monitored with SHIMADZU SPD-M20A prominence diode array detector. The column oven was set

at 40 °C. This yielded known azodyrecins A–C (**1**–**3**), 1'-*trans*-azodyrecins A–C (**4**–**6**), together with new analogs azodyrecins D–G (**7**–**10**). Following amounts of compounds were obtained from *Streptomyces* sp. RM72 grown on 11 L of YMS^{++} solid media; azodyrecin A (**1**), 1.5 mg; azodyrecin B (**2**), 2.2 mg; azodyrecin C (**3**), 1.1 mg; 1'-*trans*-azodyrecin A (**4**), 1.1 mg; 1'-*trans*-azodyrecin B (**5**), 7.9 mg; 1'-*trans*-azodyrecin C (**6**), 1.6 mg; azodyrecin D (**7**), 13.5 mg; azodyrecin E (**8**), 29.1 mg; azodyrecin F (**9**), 1.4 mg; azodyrecin G (**10**), 0.9 mg.

1'-*trans*-Azodyrecin A (**4**): colorless oil; $[\alpha]_D^{22.4}$ –4.9 (*c* 1.15, CH_3OH); ^{1}H NMR (methanol-d_4, 500 MHz) 7.09 (d, 1H, *J*=13.6), 7.02-6.96 (m, 1H), 4.50 (q, 1H, *J*=7.2), 3.68 (s, 3H), 2.27-2.23 (m, 2H), 1.55-1.46 (m, 1H), 1.49 (d, 1H, *J*=7.2), 1.40-1.25 (m, 14H), 1.20-1.14 (m, 2H), 0.87 (d, 6H, *J*=6.7), ^{13}C NMR (methanol-d_4, 125 MHz) 173.0, 138.2, 136.9, 60.2, 52.6, 40.2, 31.0-30.2, 29.4, 29.1, 23.0, 16.0; HRESI(+)MS *m/z* 349.24594 [M+Na]$^+$; calcd. For $C_{18}H_{34}N_2O_3Na^+$ 349.24616.

Azodyrecin D (**7**): colorless oil; $[\alpha]_D^{22.5}$ –14.2 (*c* 3.00, CH_3OH); NMR (methanol-d_4, 500 MHz) see Table 1; HRESI(+)MS *m/z* 351.26147 [M+Na]$^+$; calcd. For $C_{18}H_{36}N_2O_3Na^+$ 351.26181.
Azodyrecin E (**8**): colorless oil; $[\alpha]_D^{22.6}$ –21.8 (*c* 1.90, CH_3OH); NMR (methanol-d_4, 500 MHz) see Table 1; HRESI(+)MS *m/z* 365.27729 [M+Na]$^+$; calcd. For $C_{19}H_{38}N_2O_3Na^+$ 365.27746.
Azodyrecin F (**9**): colorless oil; $[\alpha]_D^{22.8}$ +0.9 (*c* 0.45, CH_3OH); NMR (methanol-d_4, 500 MHz) see Table 1; HRESI(+)MS *m/z* 379.29275 [M+ Na]$^+$; calcd. For $C_{20}H_{40}N_2O_3Na^+$ 379.29311.
Azodyrecin G (**10**): colorless oil; $[\alpha]_D^{22.6}$ –4.63 (*c* 0.35, CH_3OH); NMR (DMSO-d_6, 500 MHz) see Table 1; HRESI(+)MS *m/z* 379.29286 [M+ Na]$^+$; calcd. For $C_{20}H_{40}N_2O_3Na^+$ 379.29311.

Cytotoxic assay. In a manner analogous to the previous report,[1] the cytotoxic activities of compounds against human ovarian adenocarcinoma SKOV-3 cells, malignant pleural mesothelioma MESO-1 cells, and immortalized T lymphocyte Jurkat cells, were examined. SKOV-3 cells were cultured in Dulbecco's Modified Eagle's medium (DMEM) supplemented with 10% fetal bovine serum, penicillin (50 U/mL), and streptomycin (50 μg/mL). MESO-1 cells were cultured in RPMI1640 medium supplemented with 10% fetal bovine serum, penicillin (50 U/mL), and streptomycin (50 μg/mL). Jurkat cells were cultured in RPMI1640 medium supplemented with 10% fetal bovine serum, penicillin (50 U/mL), streptomycin (50 μg/mL), and GlutaMAX. All cell lines were seeded in a 384-well plate at a density of 1000 cells/well in 20 μL of media and incubated at 37 °C in a humidified incubator with 5% CO_2. After 4 h, 2-fold serial dilution samples dissolved in DMSO were added to the cell cultures at the concentration of 0.5% (0.1 μL) and incubated for 72 h. Cell viabilities were measured using a CellTiter-Glo luminescent cell viability assay and EnVision multilabel plate reader.

P388 murine leukemia cells were cultured in DMEM, supplemented with 1% penicillin/streptomycin and 10% fetal bovine serum in 5% of CO_2 cell incubator at 37 °C. The cells were placed a 96-well cell culture plate at a density of 1 × 10^4 cells/well, then 1 μL of test solution in various concentrations (samples were dissolved in DMSO) added to cell plates and incubated for 48 h. Doxorubicin hydrochloride was used as a positive control. Finally, 50 μL of 3-(4,5-dimethylthiazol-2-yl)-2,5-diphenyl tetrazolium bromide (MTT) solution (1 mg/mL dissolved in PBS buffer) were added to each well and the plates were incubated for 4 h. After the medium was removed, the precipitated dye was solubilized by DMSO, and measured by a microplate reader with the absorbance at 570 nm.

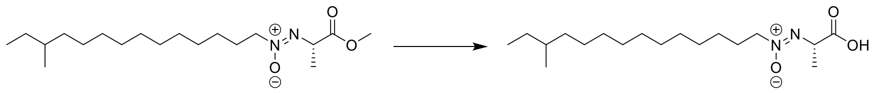

8 **11**

Preparation of demethylazodyrecin E (11). Demethyl azodyrecin E (**11**) was prepared by treating azodyrecin E (**8**) with 2 M NaOH aq. at room temperature for 20 min. The reaction mixture was acidified with 2 M acetic acid aq. until pH 7, extracted with EtOAc (3 times), and the solvent was removed. The residue was dissolved in DMSO and subjected to HPLC purification. Separation was performed using a reversed-phase column COSMOSIL 5C_{18}-MS-II 4.6 × 250 mm column (Nacalai tesque) eluted with 92% acetonitrile containing 0.1% formic acid as a mobile phase with a flow rate of 1.0 mL min^{-1}.

Demethylazodyrecin E (**11**): colorless amorphous solid; NMR (500 MHz, DMSO-d_6) δ 4.22–4.15 (m, 3H), 2.19–2.08 (m, 2H), 1.55–1.40 (m, 3H), 1.34–1.14 (m, 20H), 1.13–0.95 (m, 2H), 0.86–0.74 (m, 6H); HRESI(+)MS *m/z* 351.26117 $[M+Na]^+$; calcd. $C_{18}H_{36}N_2O_3Na^+$ 351.26181.

Cloning and expression of Ady1. *ady1* was amplified with the following oligonucleotides: Fw: 5’-ccggaattccatatgacccagaacgacg-3’ (EcoRI/NdeI sites are underlined), Rv: 5’- cggaagcttttatttccgcattccg-3’ (HindIII site is underlined), from the genomic DNA of *Streptomyces* sp. RM72 using KOD One PCR Master Mix. The amplified fragments were digested by NdeI/HindIII, then inserted into multi-cloning site (MCS) of pCold II (TaKaRa) to generate expression plasmid pCold-*ady1*. The expression plasmids were introduced into *E. coli* BL21-CodonPlus(DE3)-RIL (Agilent).

In a manner similar to the previous report,[10] a single colony of the *E. coli* hosts harboring expression plasmids was inoculated into 2xYT media containing 200 μg/mL ampicillin and 30 μg/mL chloramphenicol, then grown for overnight at 37 °C. Then, 1.0% of the overnight culture broth was transferred to 200 mL of 2xYT media containing 200 μg/mL ampicillin and 30 μg/mL chloramphenicol then cultured at 37 °C for 3 h. The expression of recombinant proteins was induced by adding IPTG at a final concentration of 0.1 mM, and cells were cultured at 16 °C for 16 h. Cells were harvested by centrifugation (3,500*g* for 10 min) and were disrupted by ultrasonic homogenizer. After removing debris by centrifugation (17,000*g* for 10 min), the supernatant was subjected to Ni-NTA affinity column (Merck Millipore) that was equilibrated by washing buffer (20 mM Tris-HCl pH 8.0, 150 mM NaCl, 20 mM imidazole). The column was washed with washing buffer and eluted with elution buffer (20 mM Tris-HCl pH 8.0, 150 mM NaCl, 500 mM imidazole). Imidazole was removed from column eluted by Amicon Ultra 0.5 mL filter (Merck Millipore). The concentration of protein solution was measured by Bio-Rad protein assay kit.

***In vitro* assay of Ady1.** 50 μL of reaction mixture containing 20 mM phosphate buffer (pH 8.0), 2.0 mM *S*-adenosylmethionine (SAM), and 0.5 mM demethylazodyrecin E (**11**) were prepared. Reaction was initiated by the addition of 5 μM recombinant Ady1, then the mixture was incubated at 30 °C for 2 h. The reaction was quenched by the addition of an equal volume of acetonitrile, centrifuged at 20,630*g* for 10 min, and the supernatants were subjected to LC–MS analysis, which was operated in the positive mode. Samples were separated by COSMOSIL 5C_{18}-MS-II (2.0 × 150 mm) (nacalai tesque) and eluted with 92% acetonitrile containing 0.1% formic acid with flow rate at 0.2 ml/min.

Bioinformatics. Functions of genes encoded in *ady* (LC712331, LC712332), biosynthetic gene cluster of KA57-A[2] and *vlm*[3] were annotated by dfast (v1.2.15)[4] using DFAST default reference database (v1.1.6) and hmmscan using Pfam-A database (35.0). HMM-based search was conducted with HMMER (3.3).[5] Protein-coding regions were predicted by prodigal (2.6.3).[6] Publicly available data were retrieved from NCBI database by using efetch (16.2) and NCBI dataset (11.22.0).
To assess the distribution of VlmA-like enzymes in publicly available database, protein sequences of 3,146 actinobacteria deposited in RefSeq database were retrieved and searched by hmmsearch using PF09924.12 (LPG_synthase_C) as a query with *E*-value at 1×10^{-10}. Proteins with more than 500 amino acids were removed by seqkit (0.15.0)[7] to give 479 sequences. Genome neighborhoods (± 20 kb) of 479 genes were scanned with phmmer with *E*-value at 1×10^{-10}, using the protein sequence of VlmH (AAN10237.1) as a query to give 179 sequences. Taxonomic information of the host organism of 179 sequence were retrieved by Taxonkit (0.8.0).[8] Four referential sequences Ady7 in RM72 (BDI55431.1), Ady7 in A1C6 (BDI55414.1), VlmA (AAN10236.1), and SRO_1850 (BBC93026.1) were added, then total 183 sequences were subjected to all-vs-all blastp (2.12.0+) with *E*-value at 1×10^{-70}. The resultant network was visualized by Cytoscape (3.9.1) (https://cytoscape.org/download.html).

Data availability. Nucleotide sequences of azodyrecins biosynthetic gene clusters were deposited under the following accession numbers.
ady in *Streptomyces* sp. RM72: LC712332
ady in *Streptomyces* sp. A1C6: LC712331

Table S1: Functional annotation of *ady* in *Streptomyces* sp. RM72 (LC712332)

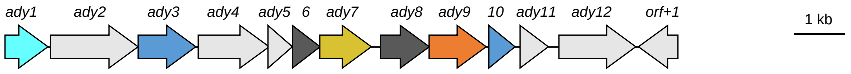

ORF	aa length	homologous protein [organism] (sequence id)	proposed function	accession no.
ady1	280	SAM-dependent methyltransferase [*Streptomyces angustmyceticus*] WP_223659814.1	Methyltransferase	BDI55425
ady2	575	putative aldehyde dehydrogenase [*Streptomyces angustmyceticus*] GES28483.1	Dehydrogenase	BDI55426
ady3	377	acyl-CoA dehydrogenase family protein [*Streptomyces angustmyceticus*] WP_152104274.1	*N*-hydroxylase	BDI55427
ady4	462	aminotransferase class III-fold pyridoxal phosphate-dependent enzyme [*Streptomyces angustmyceticus*] WP_152104273.1	Aminotransferase	BDI55428
ady5	157	SRPBCC family protein [*Streptomyces angustmyceticus*] WP_152104272.1	Unknown	BDI55429
ady6	179	hypothetical protein [*Streptomyces angustmyceticus*] WP_223659821.1	Unknown	BDI55430
ady7	338	DUF2156 domain-containing protein [*Streptomyces angustmyceticus*] WP_223659823.1	VlmA-like	BDI55431
ady8	320	VlmB-like protein [*Streptomyces angustmyceticus*] WP_086717659.1	Unknown	BDI55432
ady9	373	acyl-CoA desaturase [*Streptomyces angustmyceticus*] WP_086717661.1	Desaturase	BDI55433
ady10	172	flavin reductase family protein [*Streptomyces angustmyceticus*] WP_086717663.1	Flavin reductase	BDI55434
ady11	185	hypothetical protein [*Streptomyces angustmyceticus*] WP_086717665.1	Unknown	BDI55435
ady12	503	AarF/UbiB family protein [*Streptomyces angustmyceticus*] WP_086717668.1	Protein kinase	BDI55436
orf+1	256	alpha/beta fold hydrolase [*Streptomyces angustmyceticus*] WP_086717669.1	Hydrolase	BDI55437

Table S2: Functional annotation of *ady* in *Streptomyces* sp. A1C6 (LC712331)

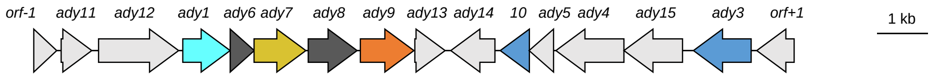

ORF	aa length	homologous protein [organism] (sequence id)	proposed function	accession no.
orf-1	149	hypothetical protein AQJ27_03030 [Streptomyces olivochromogenes] KUN49653.1	Unknown	BDI55409
ady11	208	hypothetical protein OK006_0282 [Actinobacteria bacterium OK006] KPI09619.1	Unknown	BDI55410
ady12	523	AarF/ABC1/UbiB kinase family protein [Streptomyces sp. S1D4-11] QIY99762.1	Unknown	BDI55411
ady1	308	S-adenosyl-L-methionine-dependent methyltransferase [Streptomyces mirabilis] GHD38780.1	Methyltransferase	BDI55412
ady6	157	hypothetical protein BX281_2346 [Streptomyces sp. Ag82_O1-15] PBC94436.1	Unknown	BDI55413
ady7	338	Lysylphosphatidylglycerol synthetase, domain of unknown function DUF2156 [Actinobacteria bacterium OK006] KPI09615.1	VlmA-like	BDI55414
ady8	319	VlmB-like protein [Streptomyces sp. Ag82_O1-15] WP_095851297.1	Unknown	BDI55415
ady9	350	acyl-CoA desaturase [Streptomyces sp. WAC00263] WP_181648913.1	Desaturase	BDI55416
ady13	198	hypothetical protein [Streptomyces mirabilis] WP_190168339.1	Unknown	BDI55417
ady14	289	alpha/beta fold hydrolase [Streptomyces sp. S1D4-11] QIY99768.1	Hydrolase	BDI55418
ady10	190	flavin reductase family protein [Streptomyces mirabilis] WP_075030311.1	Flavin reductase	BDI55419
ady5	160	SRPBCC family protein [Streptomyces mirabilis] WP_212726296.1	Unknown	BDI55420
ady4	444	Acetylornithine transaminase [Actinobacteria bacterium OK006] KPI09608.1	Transaminase	BDI55421
ady15	384	SDR family oxidoreductase [Streptomyces mirabilis] WP_190168343.1	Dehydrogenase	BDI55422
ady3	377	acyl-CoA/acyl-ACP dehydrogenase [Streptomyces sp. S1D4-11] QIY99772.1	*N*-hydroxylase	BDI55423
orf+1	249	hypothetical protein [Streptomyces sp. GbtcB7] WP_099919458.1	Hydrolase	BDI55424

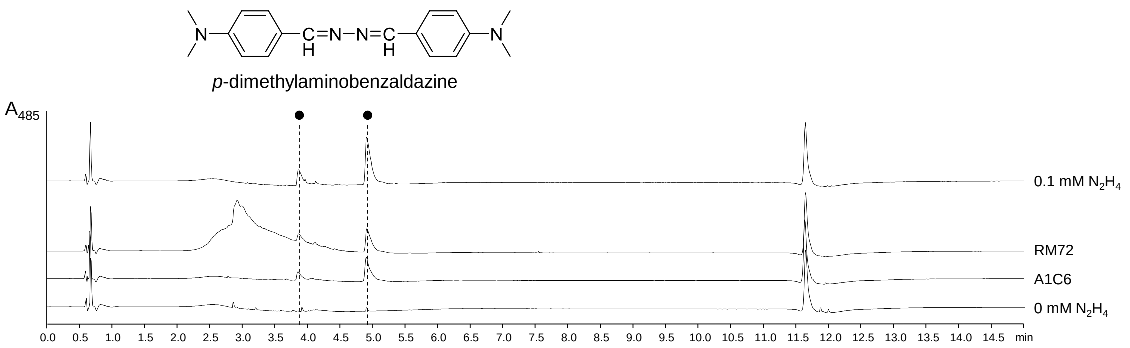

Figure S1: N_2H_4-detecting colorimetric assay of *Streptomyces* sp. RM72 and *Streptomyces* sp. A1C6. *Streptomyces* sp. RM72 and *Streptomyces* sp. A1C6 were cultured on five plates (100 mL) of SFM solid media (2.0% mannitol, 2.0% soya flour, 2.0% agar dissolved in tap water) for 7 days at 30 °C. The plates were extracted by methanol for overnight at room temperature then debris was filtered off. The filtrate was evaporated, and the residue was dissolved in 5 mL methanol. 50 µL of the supernatant were subjected to the N_2H_4-based reactivity assay following the procedure described in the previous section.

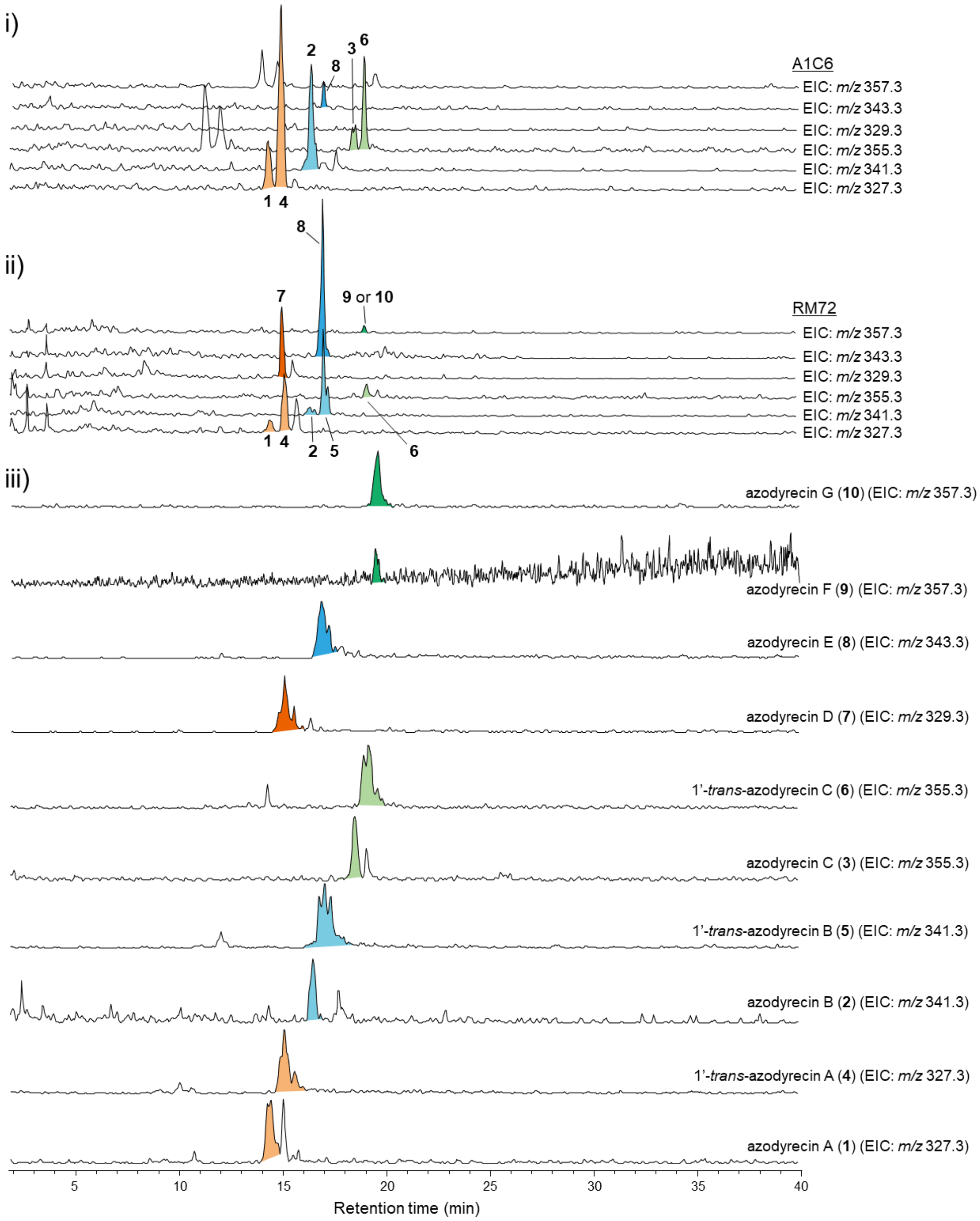

Figure S2: Production of azodyrecins by *Streptomyces* sp. RM72 and *Streptomyces* sp. A1C6. Strains were cultured on five plates (100 mL) of SFM solid media for 7 days at 30 °C. The plates were extracted by methanol for overnight at room temperature then debris was filtered off. The filtrate was evaporated, and the residue was dissolved in 5 mL methanol. 5 µL of the supernatant were subjected to LC–MS analysis. MS was operated in the positive mode. Samples were separated by COSMOSIL 5C$_{18}$-MS-II 2.0 × 100 mm. Samples were eluted by H_2O/MeCN containing 0.1% formic acid with

linear gradient: 70–98% for MeCN + 0.1% formic acid over 30 min with flow rate at 0.4 mL/min. Extracted ion chromatograms (EICs) for *m/z* 327.3 (**1**, **4**), *m/z* 341.3 (**2**, **5**), *m/z* 355.3 (**3**, **6**), *m/z* 329.3 (**7**), *m/z* 343.3 (**8**), *m/z* 357.3 (**9**, **10**) are shown. Trace i shows EIC from extracts of *Streptomyces* sp. A1C6. Trace ii shows EIC of extracts from *Streptomyces* sp. RM72. Trace iii shows EIC of standards.

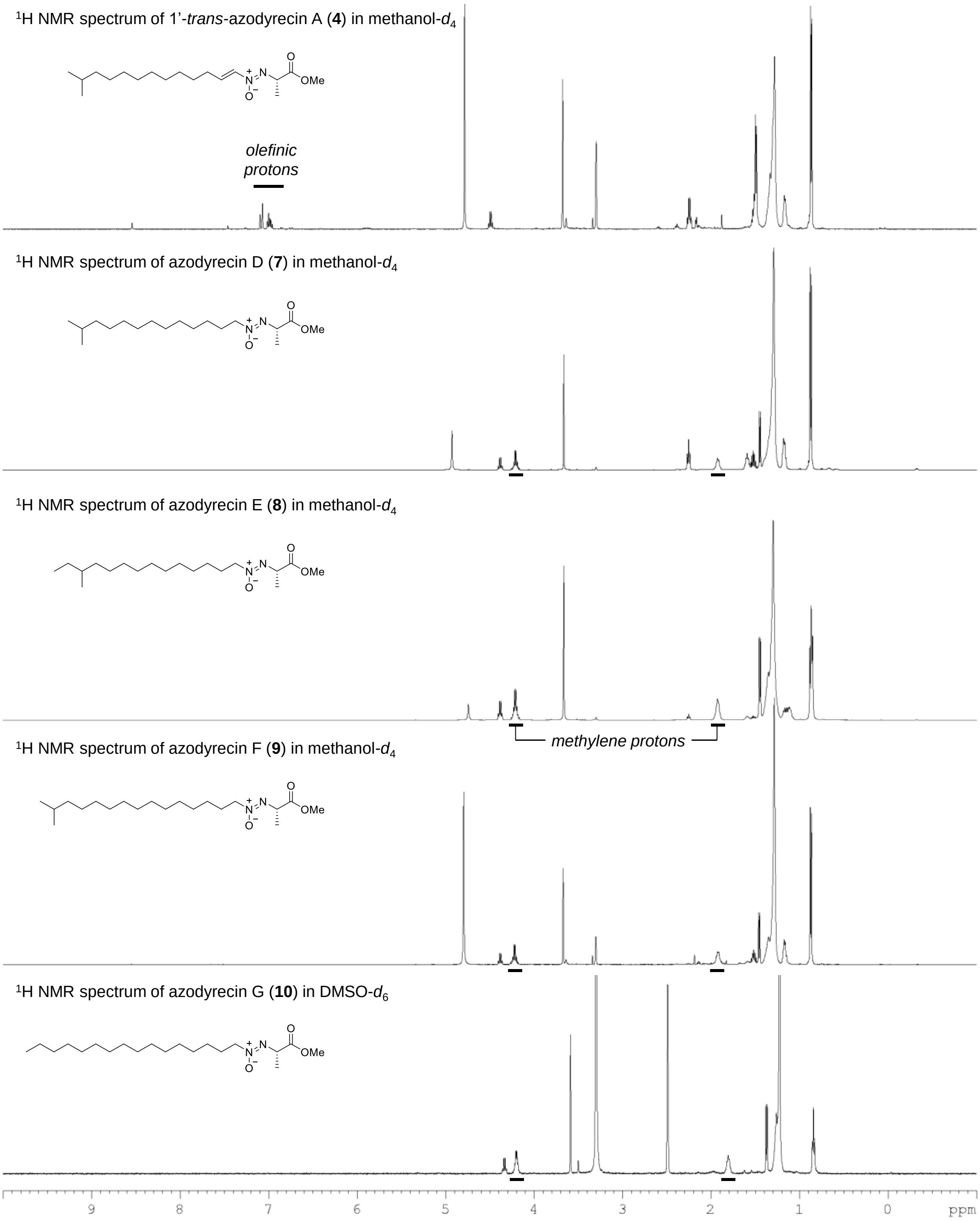

Figure S3: Comparison of ^{1}H NMR spectra of 1'-*trans*-azodyrecin A (**4**) and new azodyrecin analogs **7–10**. Compound **4** possesses the olefinic proton signals (7.09 ppm and 6.99 ppm), while compounds **7–10** possess methylene protons signals (4.21 ppm and 1.93 ppm in **7**, 4.21 ppm and 1.93 ppm in **8**, 4.22 ppm and 1.92 ppm in **9**, and 4.21 ppm and 1.81 ppm in **10**).

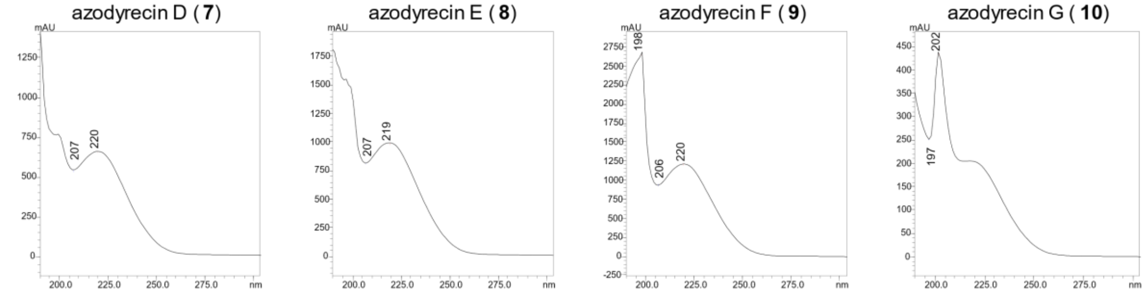

Figure S4: UV–vis spectra of azodyrecin D–G (**7**–**10**).

a)

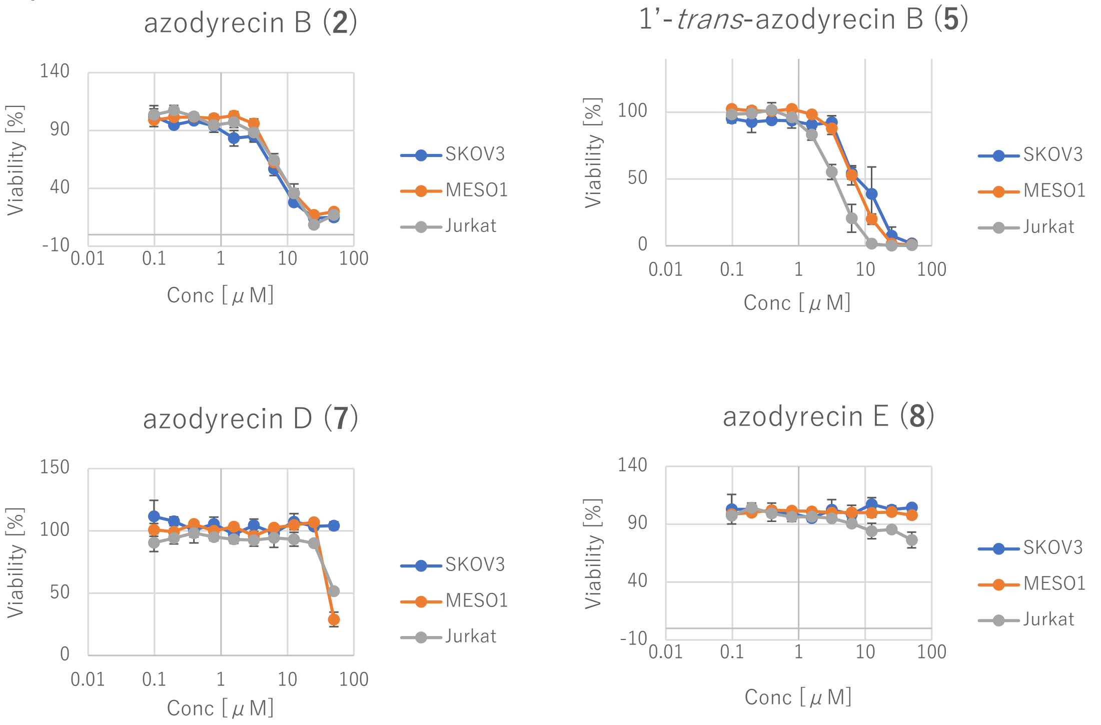

b)

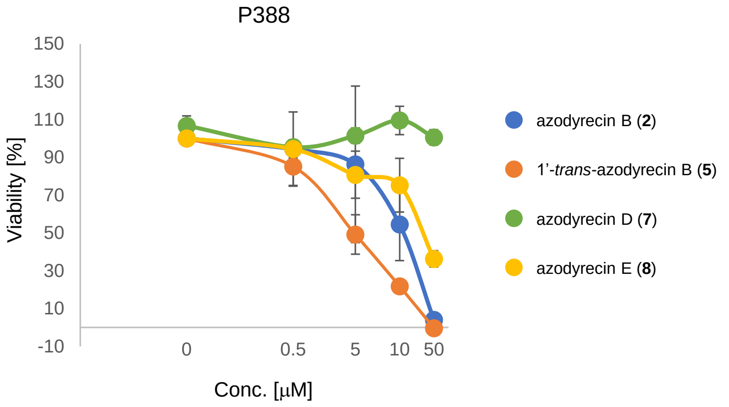

Figure S5: Cytotoxic assay of azodyrecins. Cytotoxicity of azodyrecin B (**2**), 1'-*trans*-azodyrecin B (**5**), azodyrecin D (**7**), and azodyrecin E (**8**) against (a) SKOV3, MECO1, Jurkat cell lines, and (b) P388 cells.

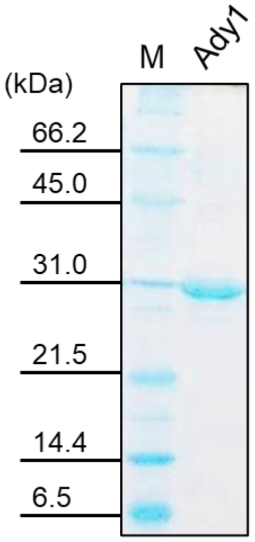

Figure S6: SDS-PAGE of Ady1. Theoretical molecular weight of the recombinant Ady1 is 31.7 kDa.

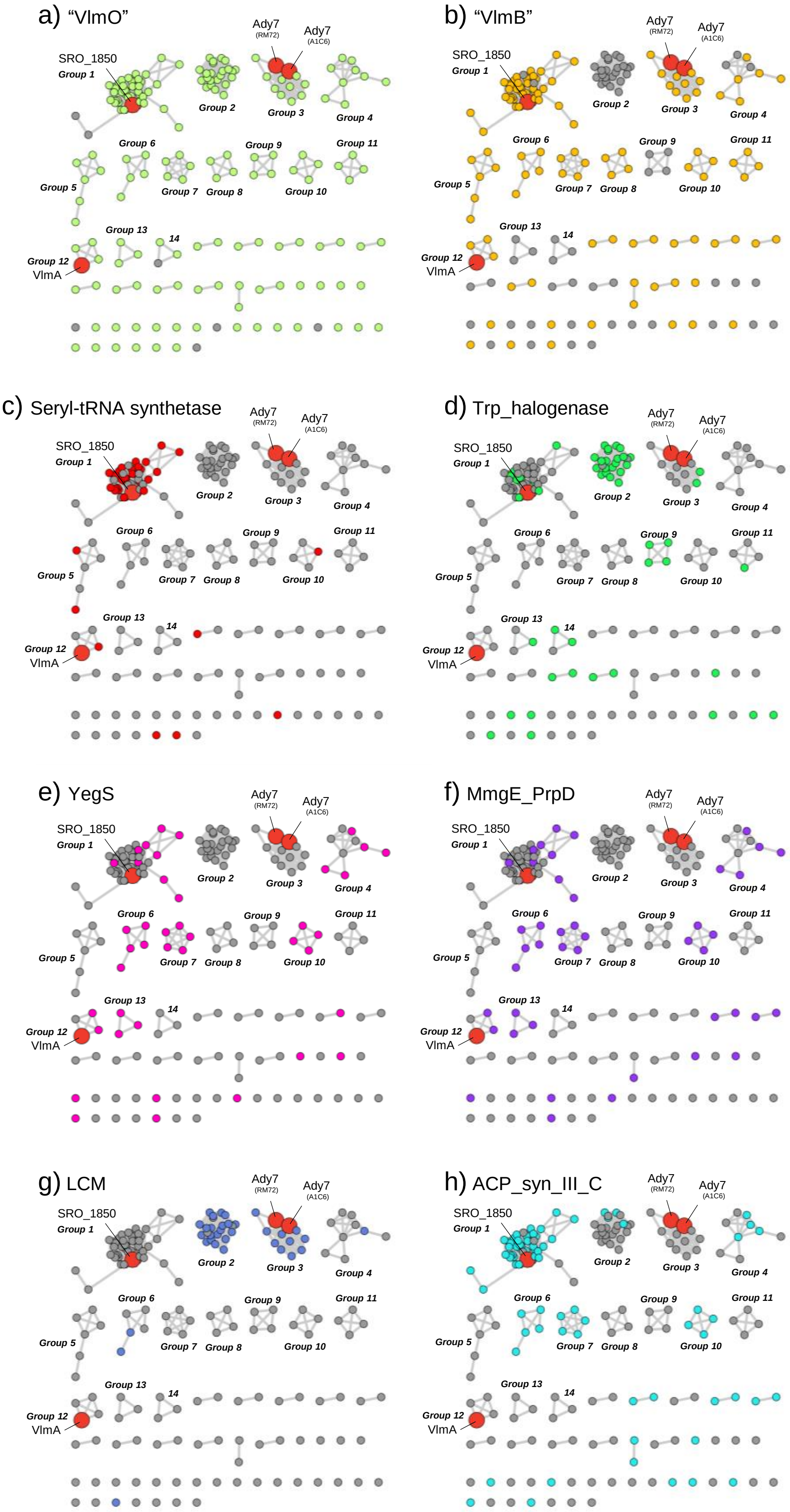
a) "VlmO"
b) "VlmB"
c) Seryl-tRNA synthetase
d) Trp_halogenase
e) YegS
f) MmgE_PrpD
g) LCM
h) ACP_syn_III_C
Ady7
(RM72)
Ady7
(A1C6)
SRO_1850
Group 1
Group 2
Group 3
Group 4
Group 5
Group 6
Group 7
Group 8
Group 9
Group 10
Group 11
Group 12
VlmA
Group 13
14

Figure S7: Protein families that are frequently co-occurring with “VlmA”. SSN of VlmA homologs (Figure 5) was colored according to the presence of the specific proteins encoded in the genome neighborhoods (± 20 kb). Genome neighborhoods were scanned by HMM-based methods, using parameters set at default. (a) “VlmA” co-occurring with “VlmO”. “VlmO” was searched by phmmer using VlmO (AAN10238.1) as a query. (b) “VlmA” co-occurring with “VlmB”. “VlmB” was searched by phmmer using VlmB (AAN10240.1) as a query. (c) “VlmA” co-occurring with seryl-tRNA synthetases. Seryl-tRNA synthetase was searched by hmmsearch using PF02403.25 (Seryl-tRNA_N) as a query. (d) “VlmA” co-occurring with tryptophan halogenases. Tryptophan halogenase was searched by hmmsearch using PF04820.17 (Trp_halogenase) as a query. (e) “VlmA” co-occurring with VlmJ-like kinases. VlmJ-like kinase was searched by hmmsearch using PF19279.2 (VegS_C) as a query. (f) “VlmA” co-occurring with VlmK-like dehydratases. VlmK-like dehydratase was searched by hmmsearch using PF03972.17 (MmgE_PrpD) as a query. (g) “VlmA” co-occurring with Ady1-like methyltransferases. Ady1-like methyltransferase was searched by hmmsearch using PF04072.17 (LCM) as a query. (h) “VlmA” co-occurring with 3-Oxoacyl-[acyl-carrier-protein (ACP)] synthase III-like enzymes. 3-Oxoacyl-[acyl-carrier-protein (ACP)] synthase III-like enzymes were searched by hmmsearch using PF08541.13 (ACP_syn_III_C) as a query.

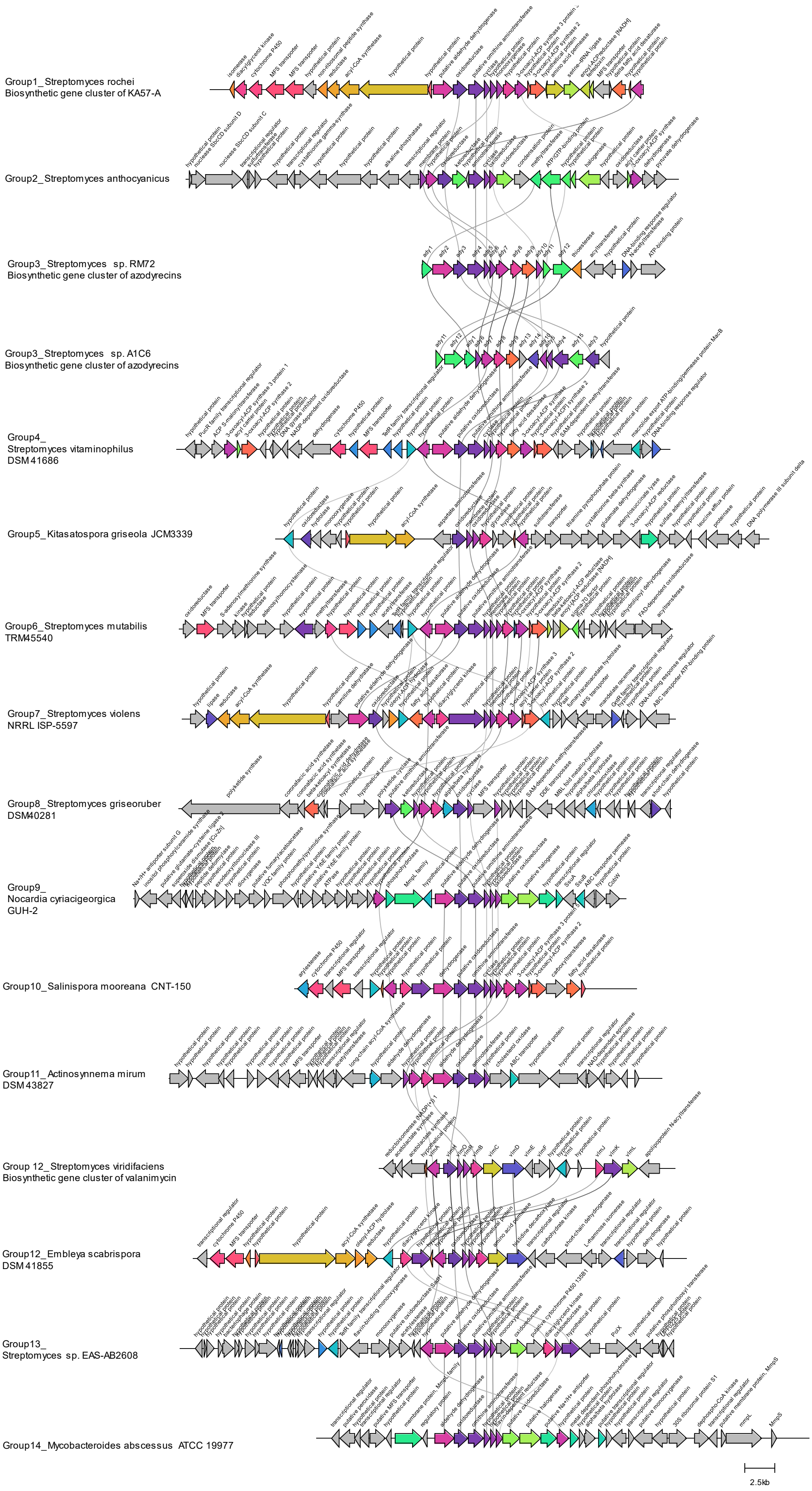
Group1_Streptomyces rochei
Biosynthetic gene cluster of KA57-A
Group2_Streptomyces anthocyanicus
Group3_Streptomyces sp. RM72
Biosynthetic gene cluster of azodyrecins
Group3_Streptomyces sp. A1C6
Biosynthetic gene cluster of azodyrecins
Group4_
Streptomyces vitaminophilus
DSM41686
Group5_Kitasatospora griseola JCM3339
Group6_Streptomyces mutabilis
TRM45540
Group7_Streptomyces violens
NRRL ISP-5597
Group8_Streptomyces griseoruber
DSM40281
Group9_
Nocardia cyriacigeorgica
GUH-2
Group10_Salinispora mooreana CNT-150
Group11_Actinosynnema mirum
DSM43827
Group 12_Streptomyces viridifaciens
Biosynthetic gene cluster of valanimycin
Group12_Embleya scabrispora
DSM41855
Group13_
Streptomyces sp. EAS-AB2608
Group14_Mycobacteroides abscessus ATCC 19977
2.5kb

Figure S8: Comparison of representative biosynthetic gene clusters of aliphatic azodyrecins. Group numbers are corresponding to those in Figure S7. The Figure was generated by clinker.[9]

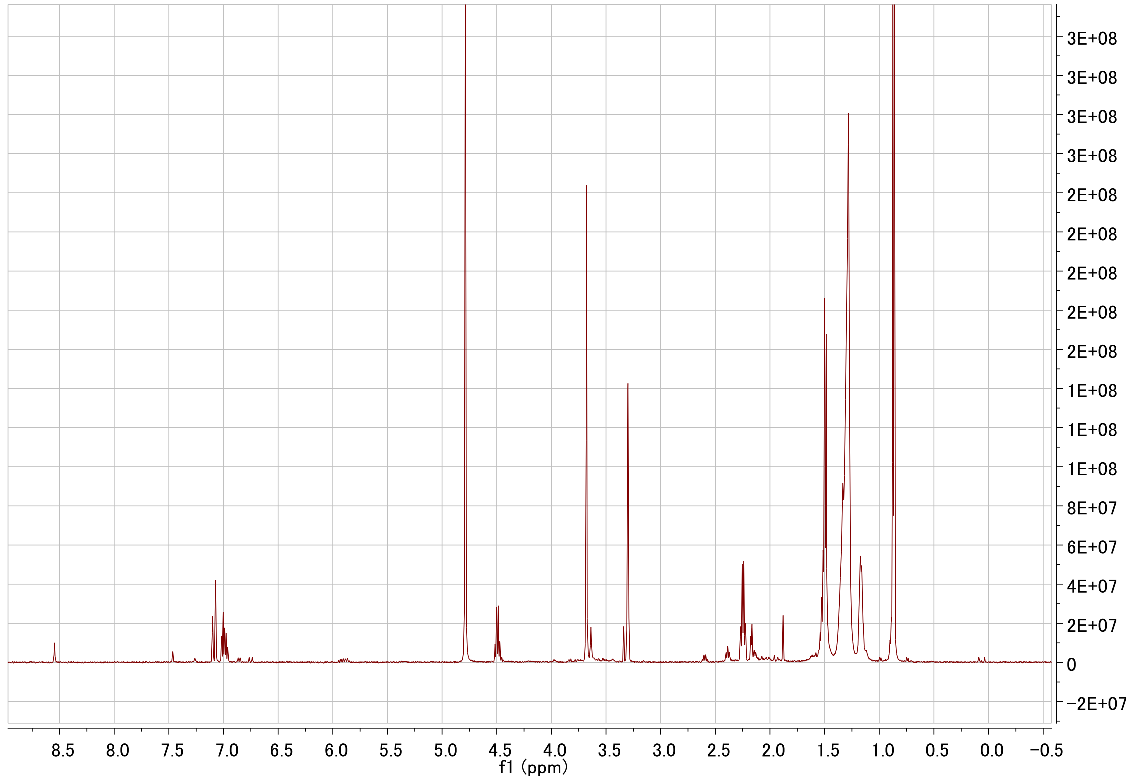

Figure S9: ^{1}H NMR spectrum of **4** in methanol-d_4.

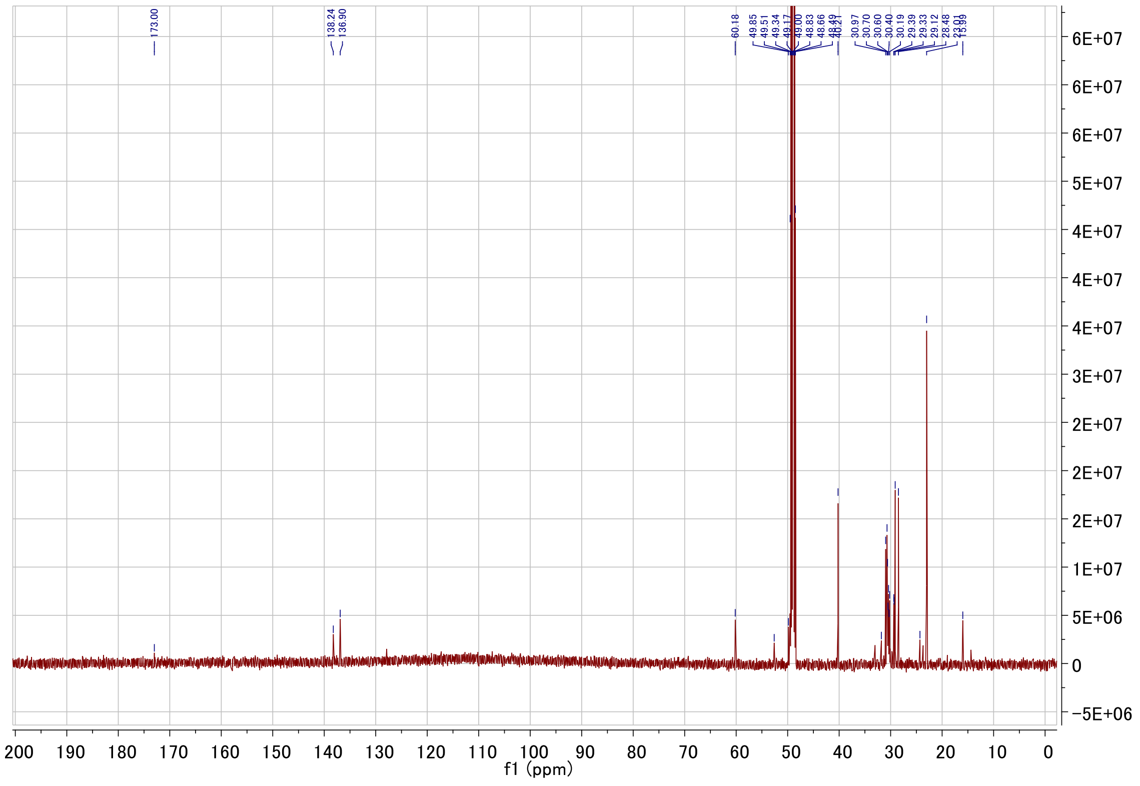

Figure S10: ^{13}C NMR spectrum of **4** in methanol-d_4.

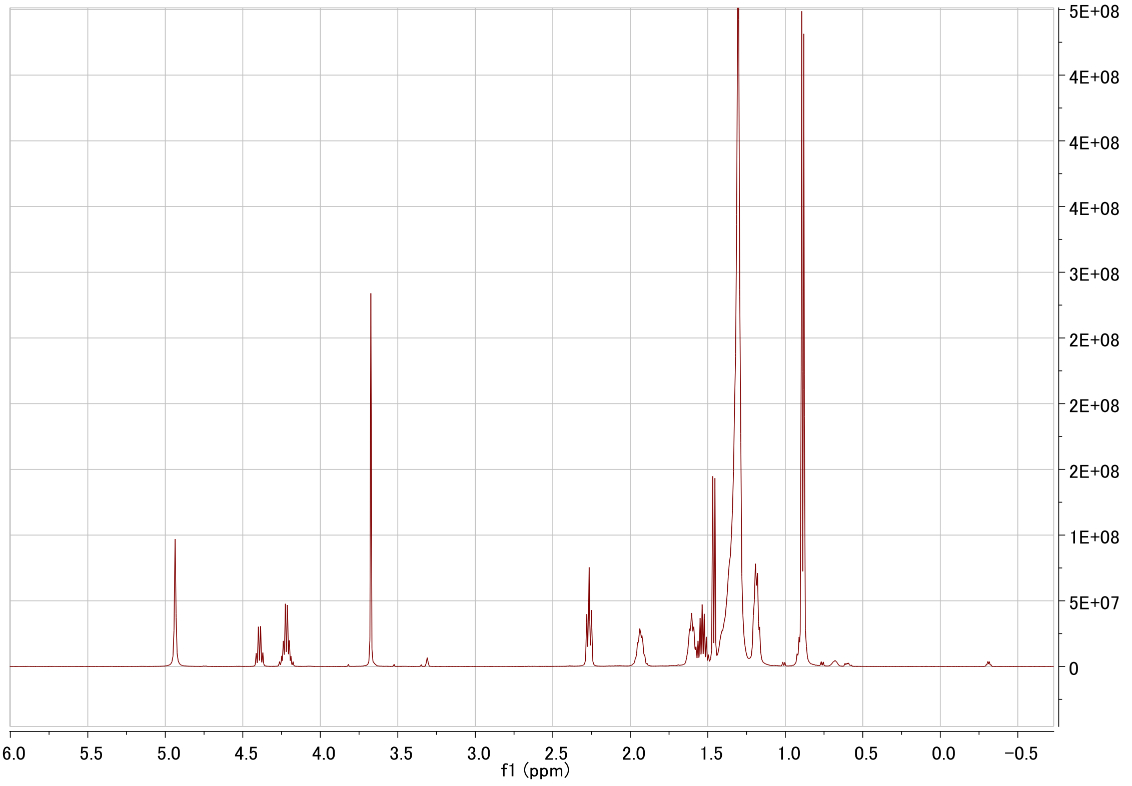

Figure S11. ^{1}H NMR spectrum of **7** in methanol-d_4.

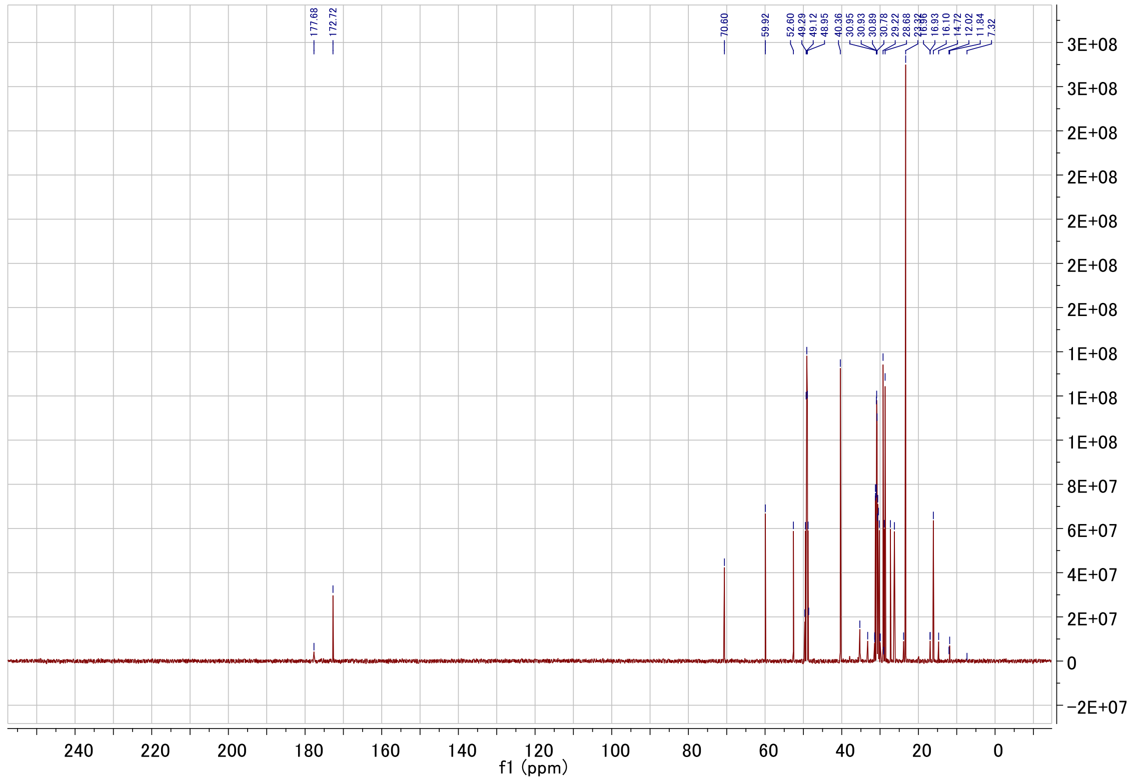

Figure S12: ^{13}C NMR spectrum of **7** in methanol-d_4.

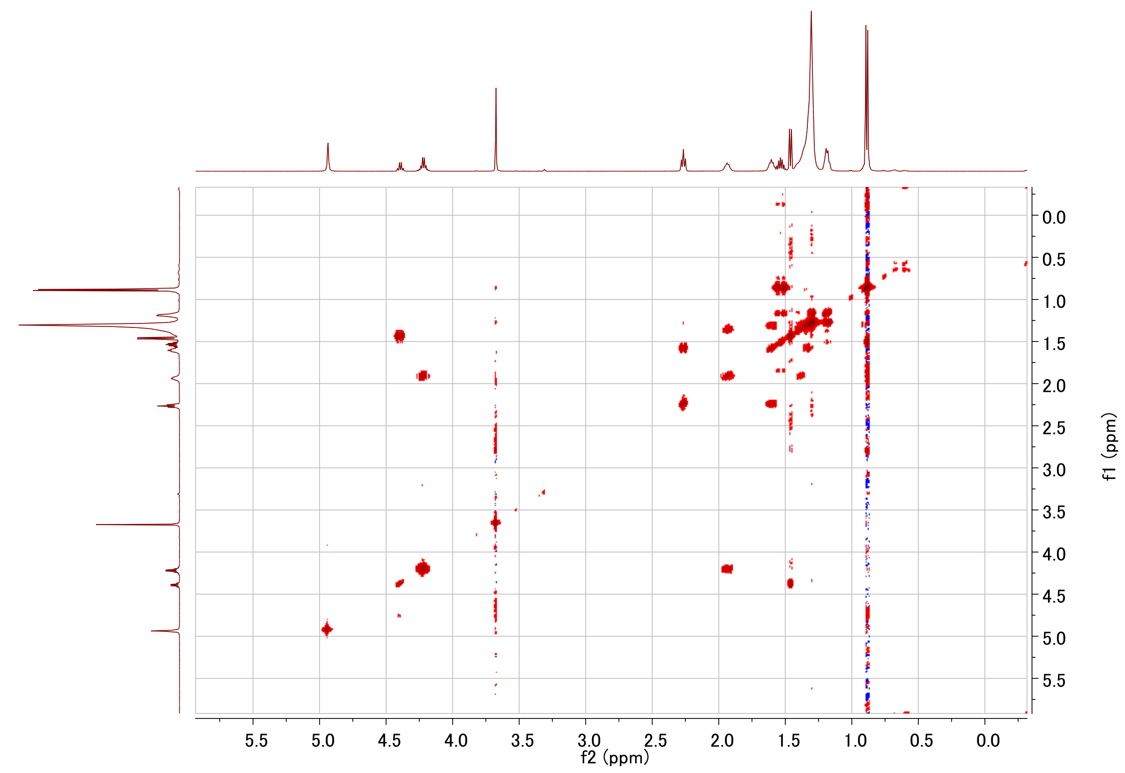

Figure S13: DQF-COSY spectrum of **7** in methanol-d_4.

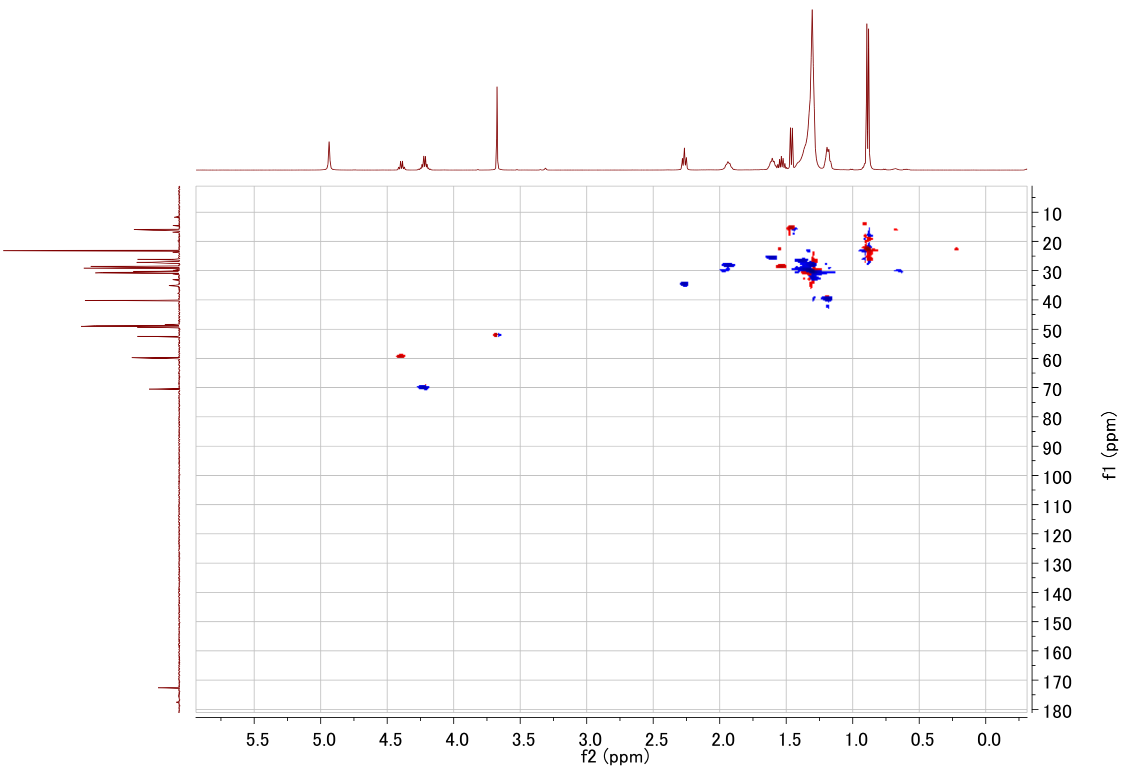

Figure S14: HSQC spectrum of **7** in methanol-d_4.

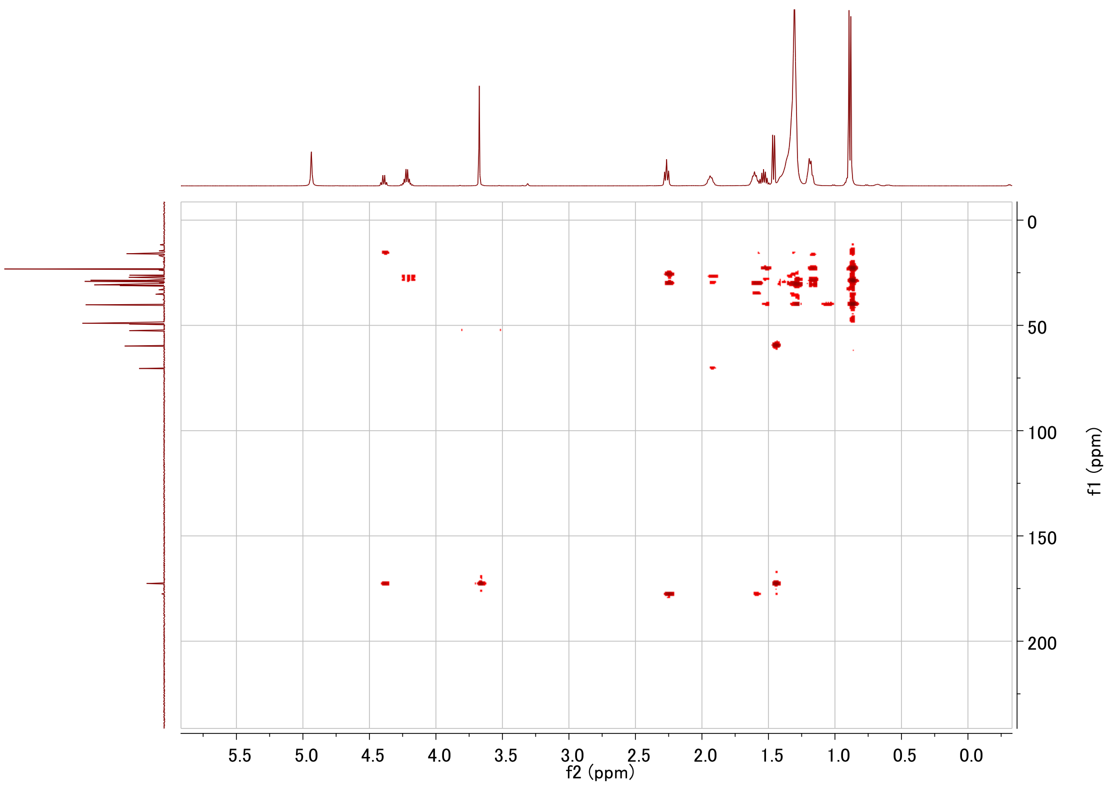

Figure S15: ^{1}H-^{13}C HMBC spectrum of **7** in methanol-d_4.

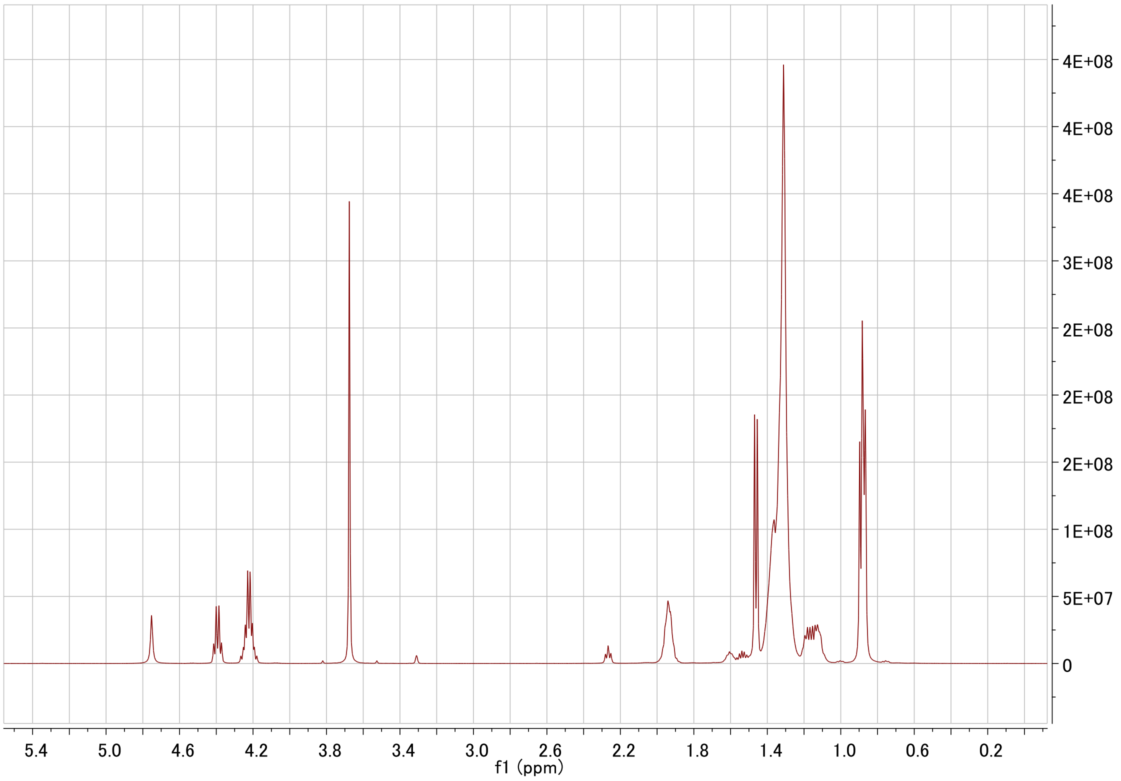

Figure S16: ^{1}H NMR spectrum of **8** in methanol-d_4.

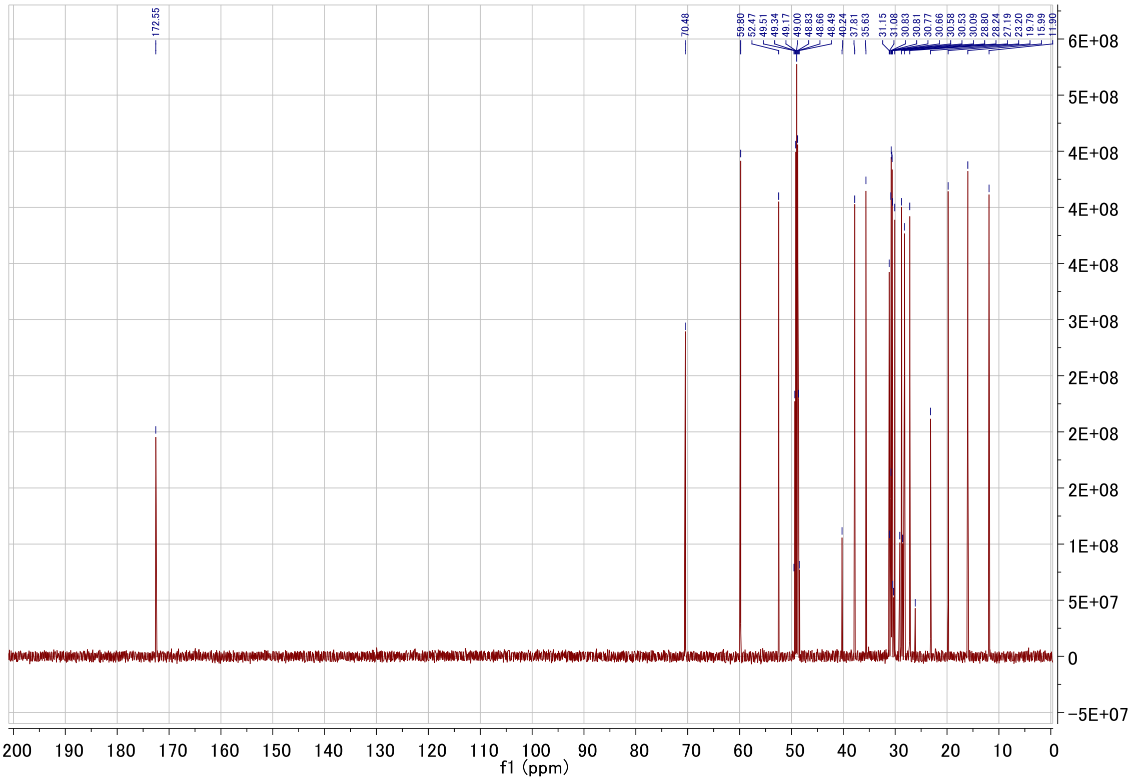

Figure S17: ^{13}C NMR spectrum of **8** in methanol-d_4.

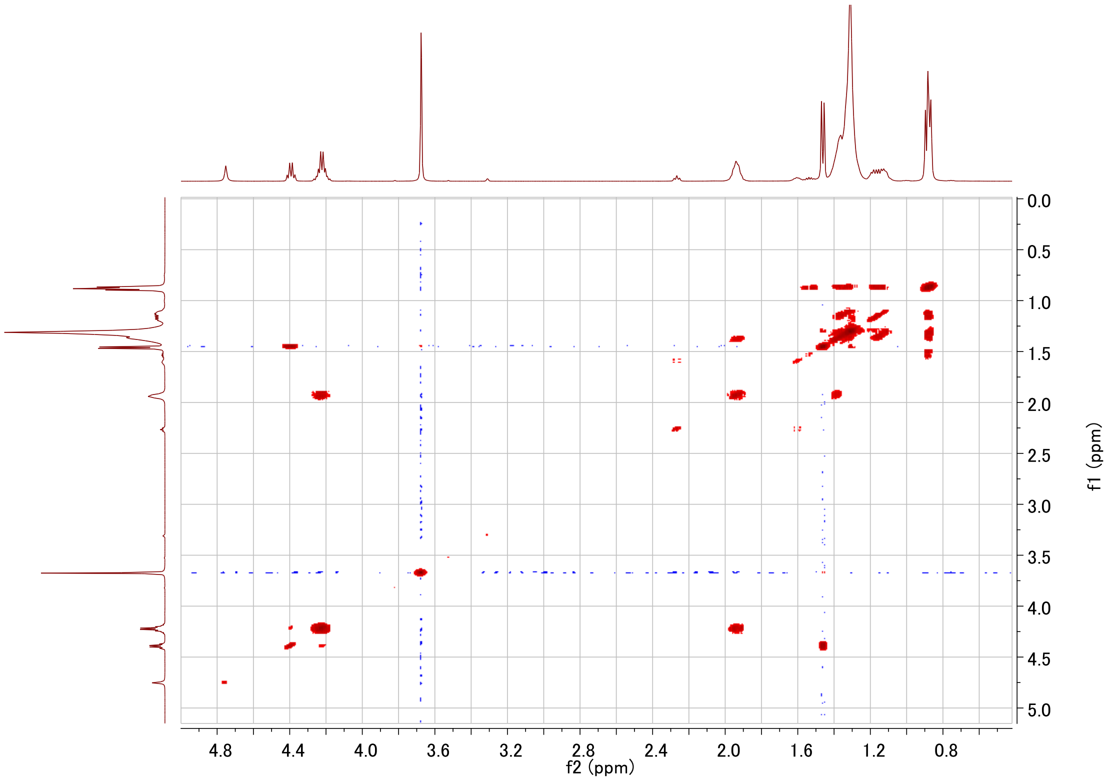

Figure S18: DQF-COSY spectrum of **8** in methanol-d_4.

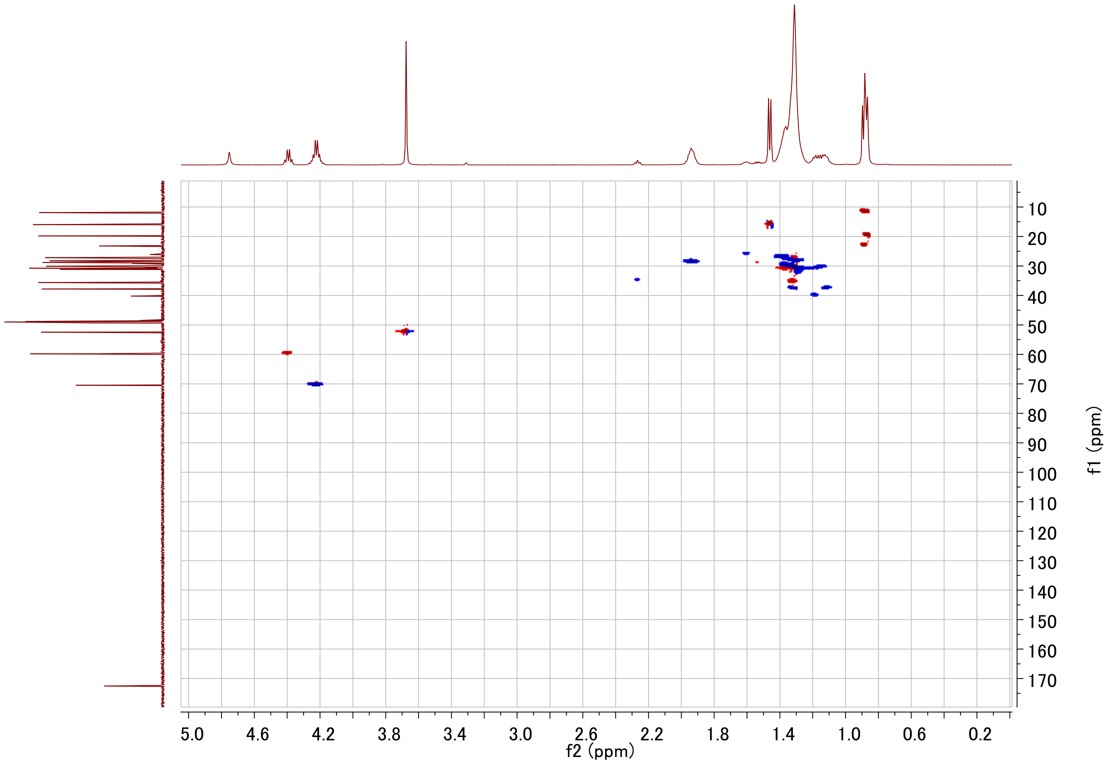

Figure S19: HSQC spectrum of **8** in methanol-d_4.

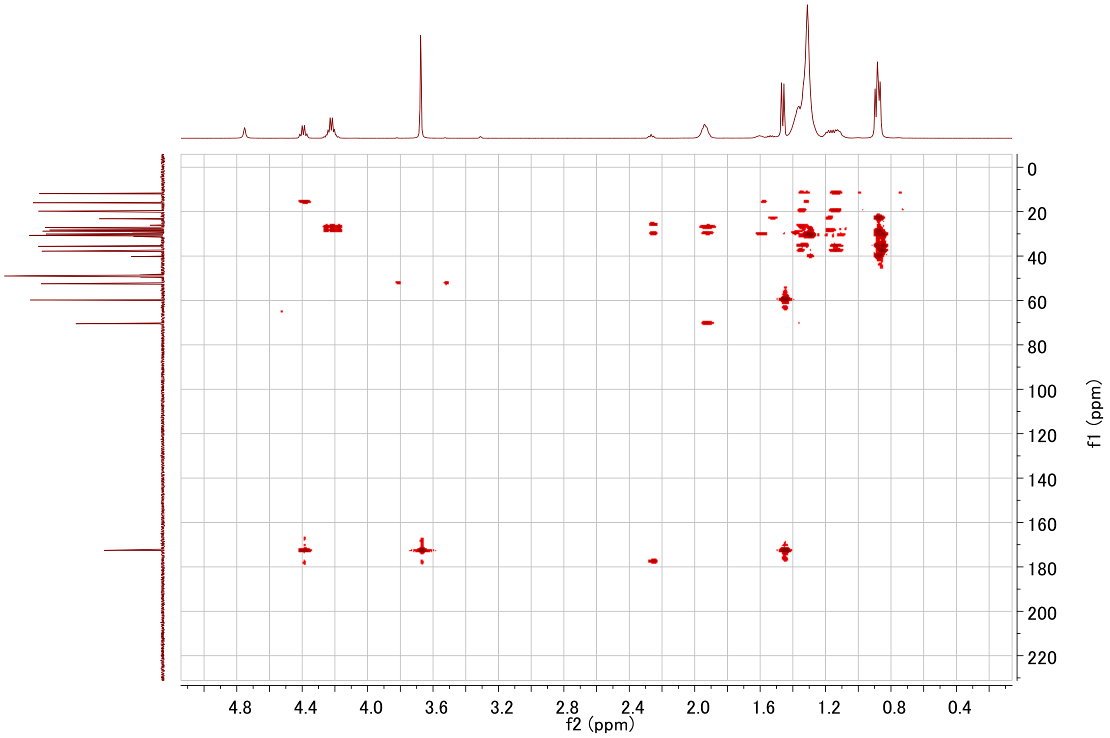

Figure S20: ^{1}H-^{13}C HMBC spectrum of **8** in methanol-d_4.

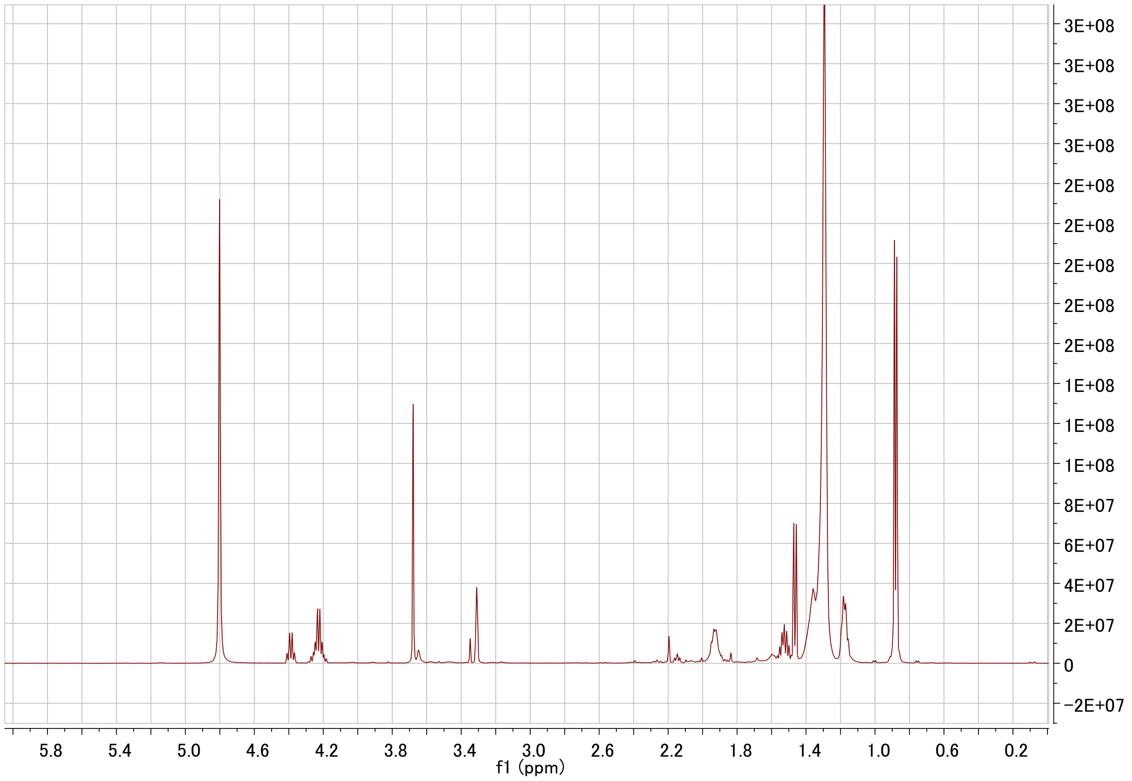

Figure S21: ^{1}H NMR spectrum of **9** in methanol-d_4.

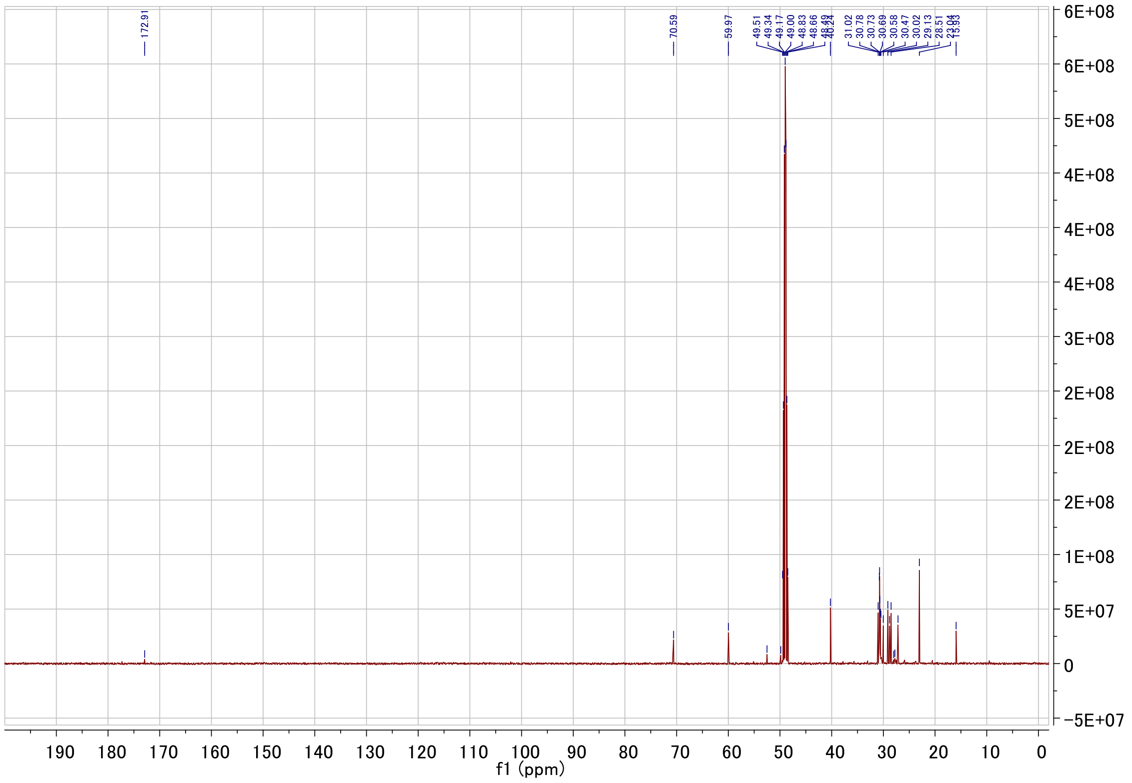

Figure S22: ^{13}C NMR spectrum of **9** in methanol-d_4.

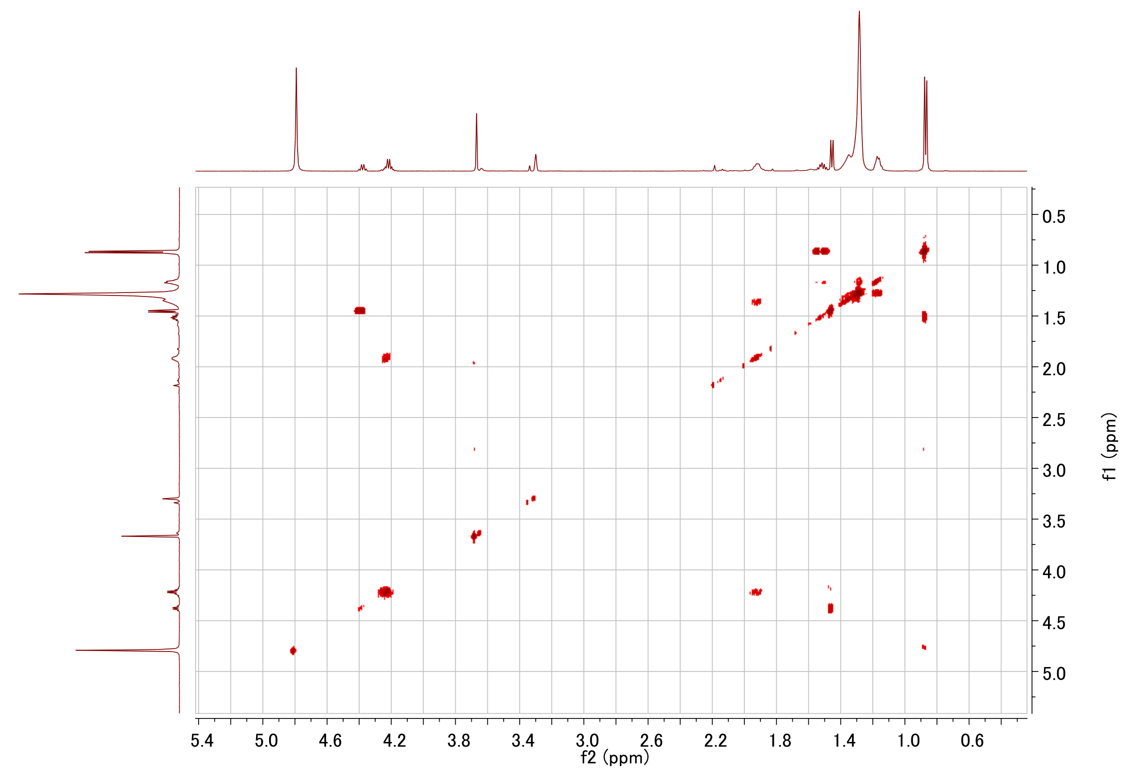

Figure S23: DQF-COSY spectrum of **9** in methanol-*d*4.

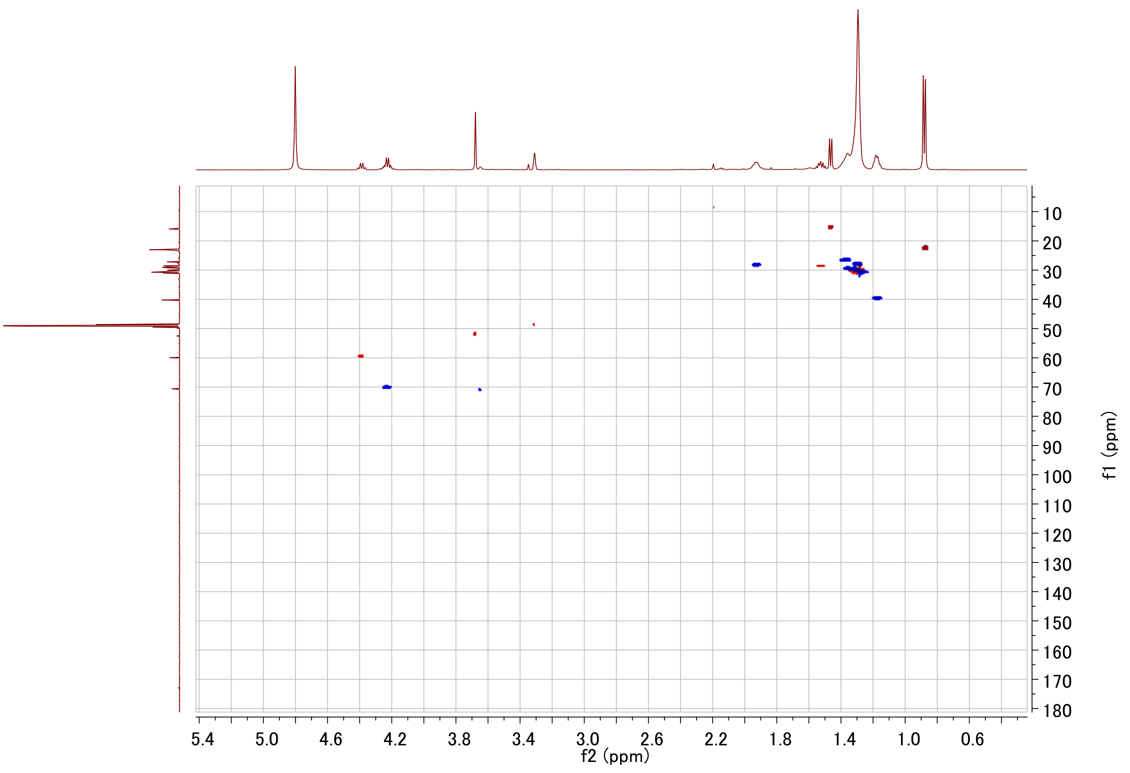

Figure S24: HSQC spectrum of **9** in methanol-*d*4.

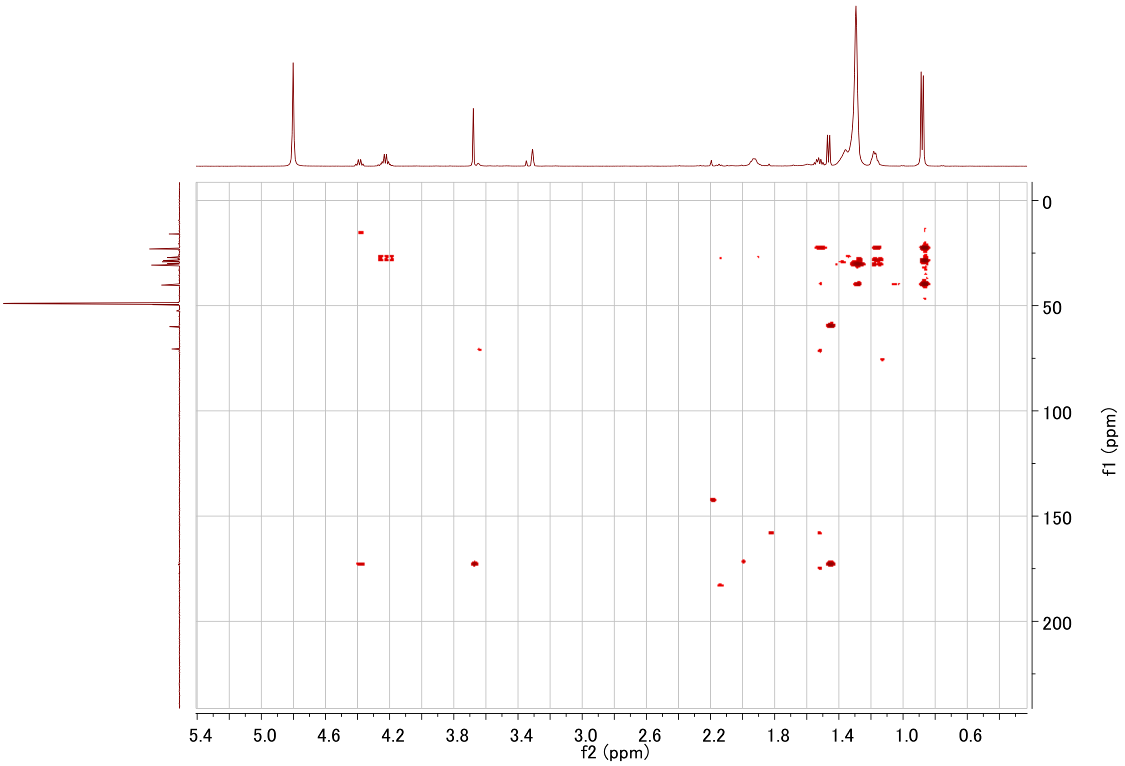

Figure S25: ^{1}H-^{13}C HMBC spectrum of **9** in methanol-d_4.

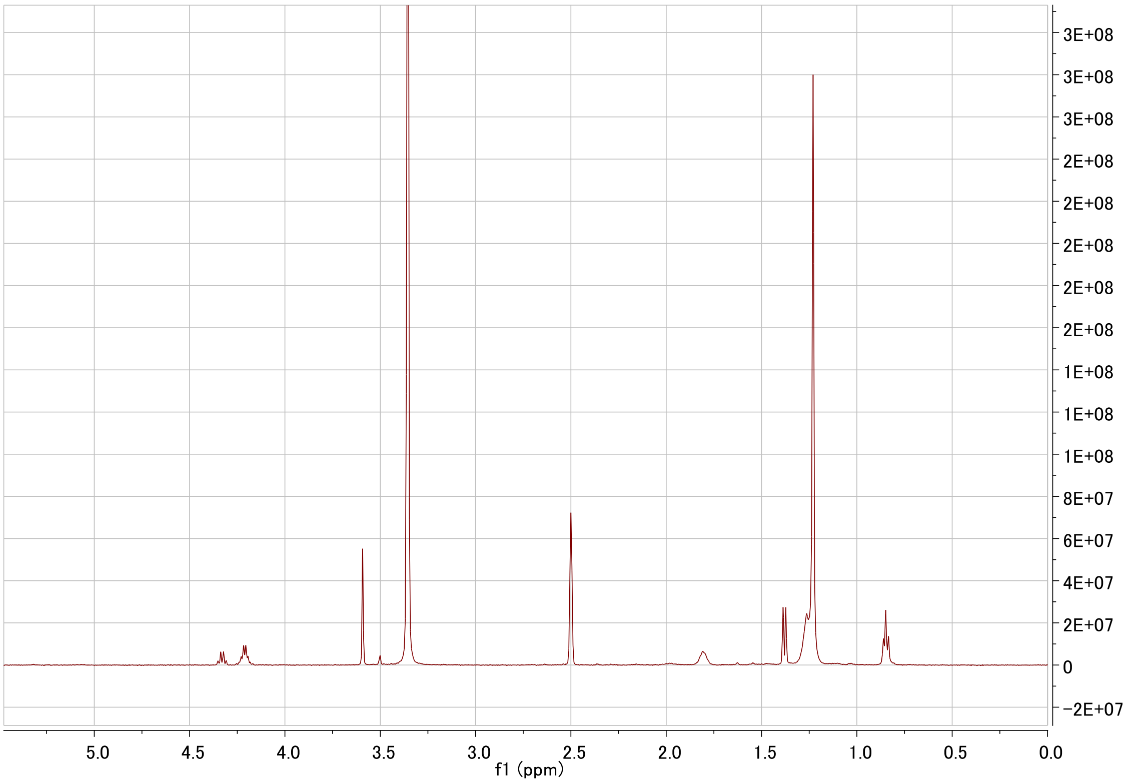

Figure S26: ^{1}H NMR spectrum of **10** in DMSO-d_6.

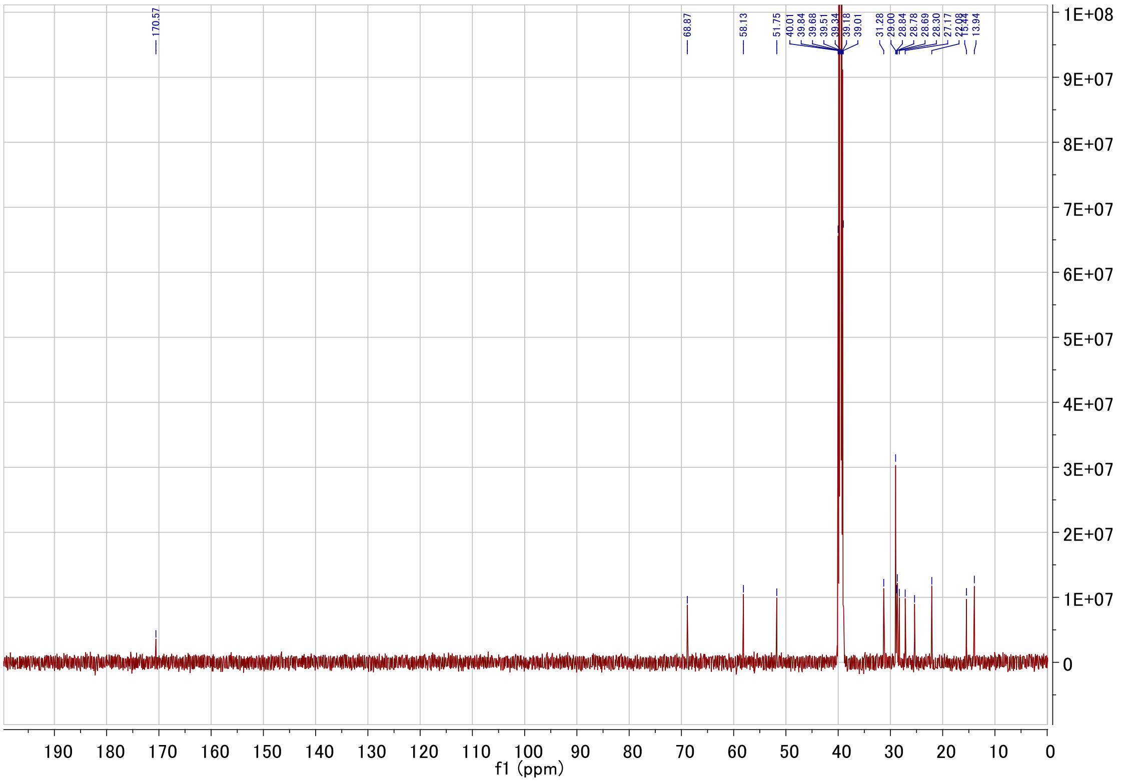

Figure S27: ^{13}C NMR spectrum of **10** in DMSO-d_6.

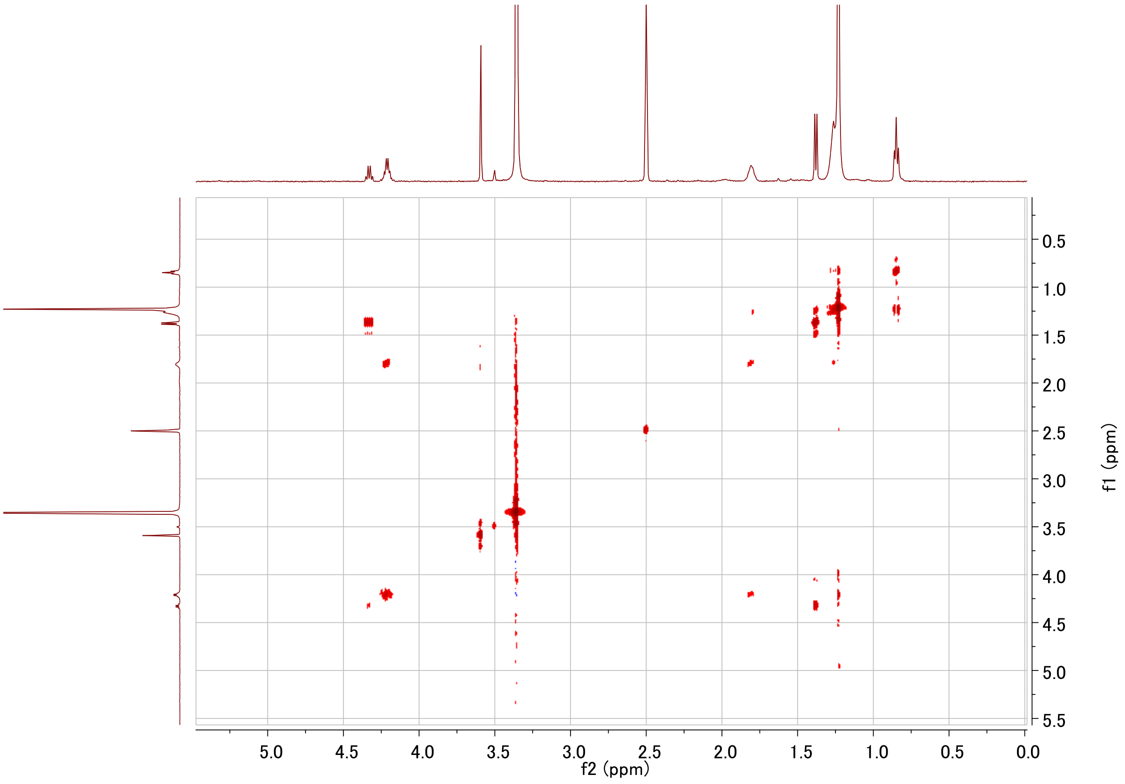

Figure S28: DQF-COSY spectrum of **10** in DMSO-d_6.

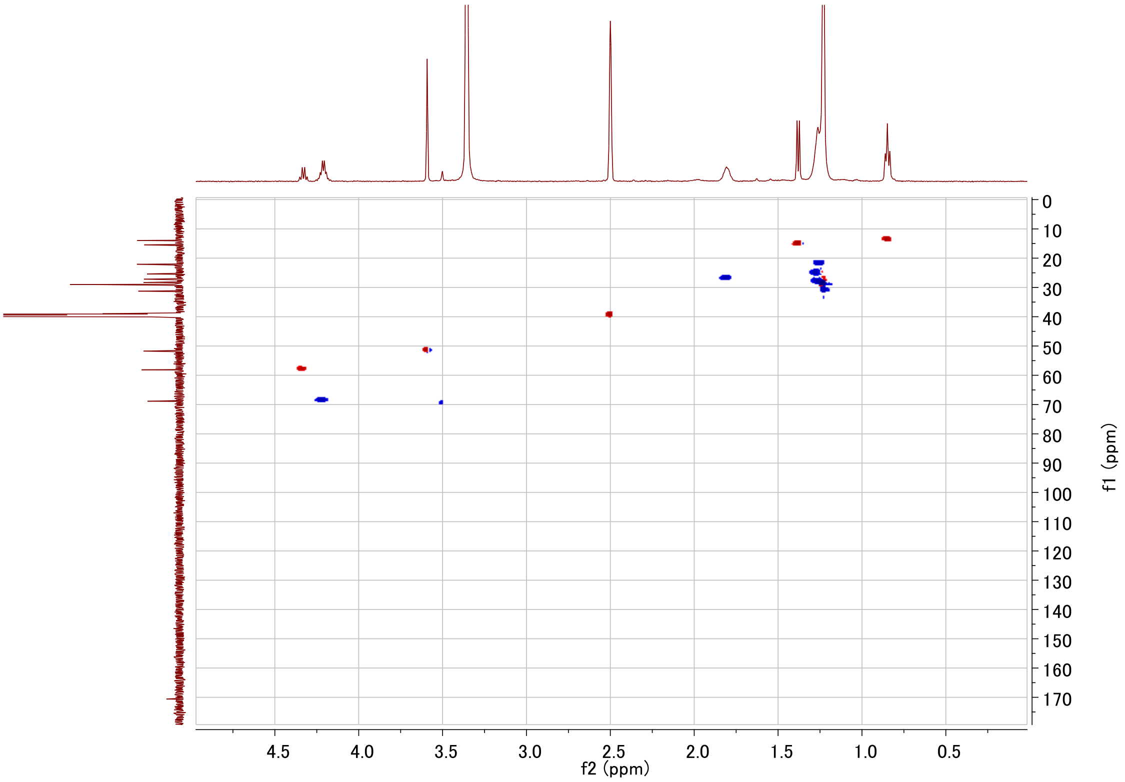

Figure S29: HSQC spectrum of **10** in DMSO-d_6.

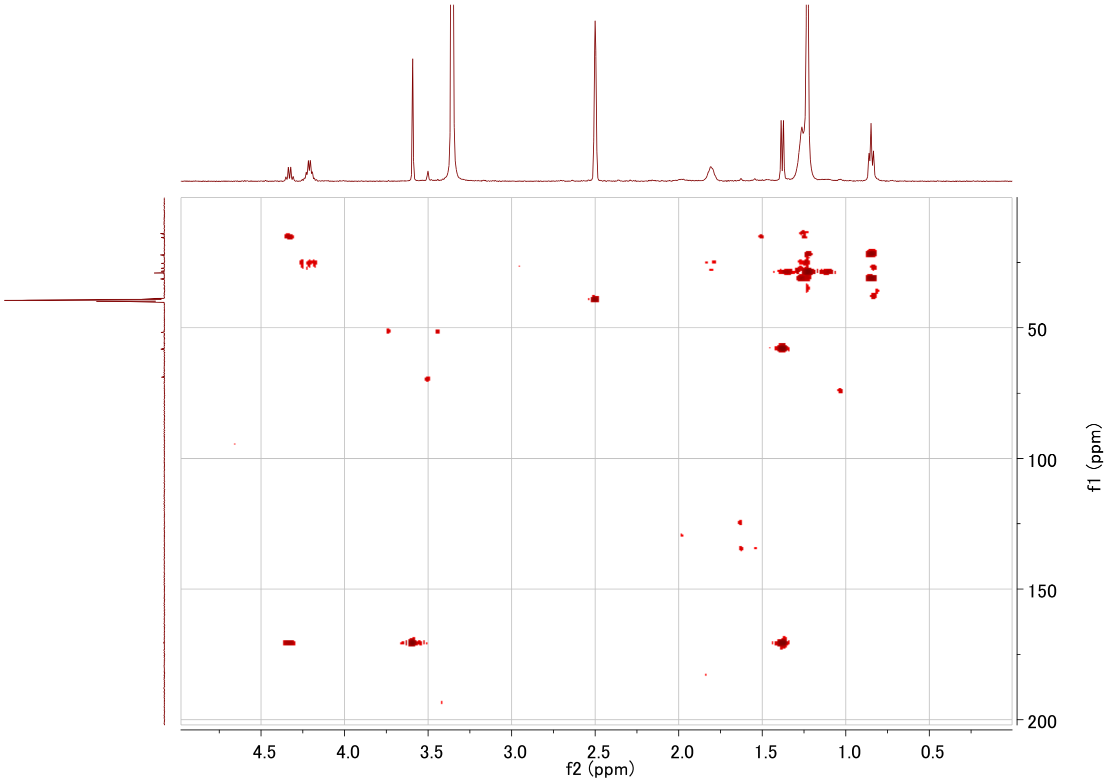

Figure S30: ^{1}H-^{13}C HMBC spectrum of **10** in DMSO-d_6.

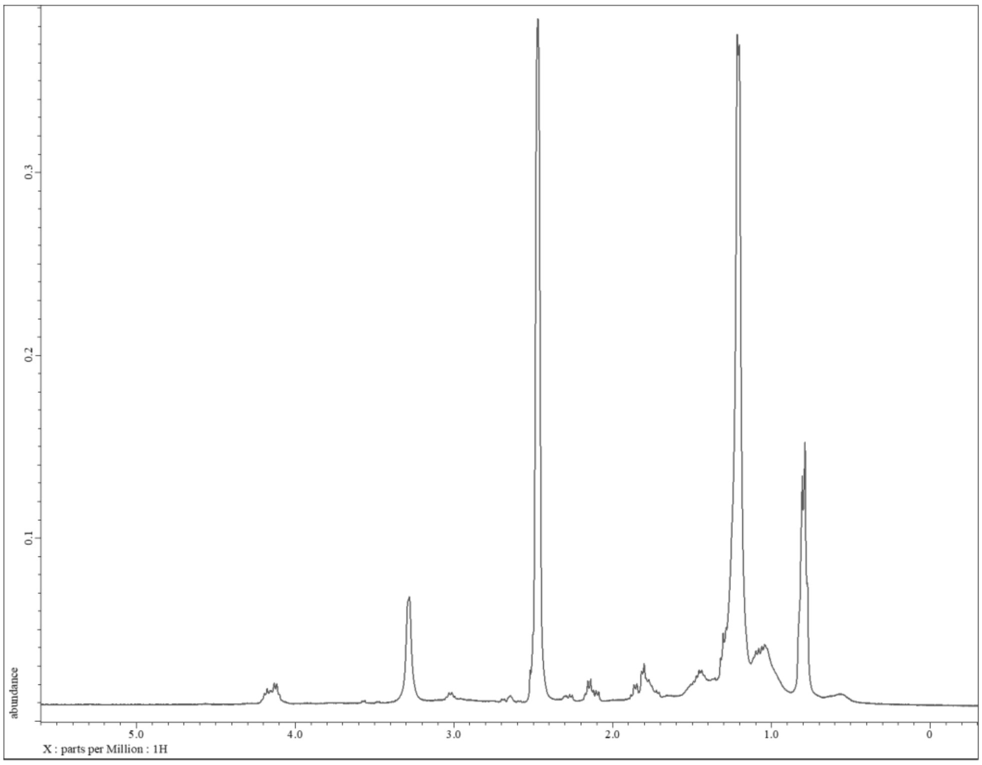

Figure S31: ^{1}H NMR spectrum of **11** in DMSO-d_6. *: Solvent peaks (methanol: δ 3.28, DMSO: δ 2.47).

References

1. Kawahara, T.; Izumikawa, M.; Kozone, I.; Hashimoto, J.; Kagaya, N.; Koiwai, H.; Komatsu, M.; Fujie, M.; Sato, N.; Ikeda, H.; Shin-Ya, K. *J. Nat. Prod.* **2018**, *81*, 264–269.
2. Nindita, Y.; Cao, Z.; Fauzi, A. A.; Teshima, A.; Misaki, Y.; Mus-limin, R.; Yang, Y.; Shiwa, Y.; Yoshikawa, H.; Tagami, M.; Lezhava, A.; Ishikawa, J.; Kuroda, M.; Sekizuka, T.; Inada, K.; Kinashi, H.; Arakawa, K. *Sci. Rep.* **2019**, *9*, 10973.
3. Garg, R. P.; Ma, Y.; Hoyt, J. C.; Parry, R. J. *Mol. Microbiol.* **2002**, *46*, 505–517.
4. Tanizawa, Y.; Fujisawa, T.; Nakamura, Y. *Bioinformatics* **2018**, *34*, 1037–1039.
5. Eddy, S. R. *PLoS Comput. Biol.* **2011**, *7*, e1002195.
6. Hyatt, D.; Chen, G. L.; Locascio, P. F.; Land, M. L.; Larimer, F. W.; Hauser, L. J. *BMC Bioinformatics* **2010**, *11*, 119.
7. Shen, W.; Le, S.; Li, Y.; Hu, F. *PLoS One* **2016**, *11*, e0163962.
8. Shen, W.; Ren, H. *J. Genet. Genomics* **2021**, *48*, 844–850.
9. Gilchrist, C. L. M.; Chooi, Y. H. *Bioinformatics* **2021**, *37*, 2473–2475.
10. Phan,C.; Matsuda, K.; Balloo, N.; Fujita, K.; Wakimoto, T.; Okino, T. *J. Am. Chem. Soc.* **2021**, *143*, *27*, 10083–10087.
